# Estrogen enhances the proliferation, migration, and invasion of papillary thyroid carcinoma via the ERα/KRT19 signaling axis

**DOI:** 10.1007/s40618-024-02473-5

**Published:** 2024-10-25

**Authors:** Z. M. Song, Y. D. Wang, F. Chai, J. Zhang, S. Lv, J. X. Wang, Y. Xi

**Affiliations:** 1 Department of Endocrinology and Metabolism, The Third Affiliated Hospital of Jinzhou Medical University, No. 2, Section 5, Heping Road, Linghe District, Jinzhou, Liaoning Province 121002 China; 2 Thyroid Surgery Department, The First Affiliated Hospital of Jinzhou Medical University, Jinzhou, China; 3 Department of Hematology, The First Affiliated Hospital of Jinzhou Medical University, Jinzhou, China; 4 Changchun Infection Disease Hospital, Changchun, China; 5 Shenbei New District Health and Wellness Supervision Center, Shenyang, China

**Keywords:** Papillary thyroid carcinoma, Estrogen, TCGA, KRT19, ERα/KRT19 signaling axis, ONT sequencing

## Abstract

**Background:**

Estrogen is thought to be the reason for the higher prevalence of papillary thyroid carcinoma (PTC) in fertile women; however, more study is required to completely comprehend how estrogen affects PTC development at the cellular level. Therefore, we combined Oxford Nanopore Technologies (ONT) sequencing to explore molecular markers of PTC and to investigate the molecular mechanisms by which estrogen promotes PTC development.

**Methods:**

The expression levels of ESR1 (ERα) and KRT19 in normal thyroid tissues and cancer tissues as well as in different cancer stages, races, genders, age groups, histological subtypes and nodular metastasis status of the TCGA database were analyzed online by Ualcan; the relationship between ESR1, KRT19 and the survival of THCA patients was analyzed. A PTC xenograft tumor model was established. An ERα specific inhibitor (MPP) was administered and an EDU cell proliferation assay was used to verify the effect of estrogen on PTC proliferation. KRT19 was knocked down in KTC-1 cells, and the proliferation, migration, and invasion abilities of PTC cells were determined using CCK-8, immunofluorescence labeling, Western blot for EMT-related proteins, scratch assay, and Transwell assay. The role of ERα in relation to KRT19 was investigated by Western blot and immunofluorescence. The effects of ERα/KRT19 signaling axis on the proliferation, migration and invasion ability of PTC cells were evaluated using EDU cell proliferation assay and Transwell. Using ONT sequencing, 15 pairs of PTC tissue and paracancer tissue samples were collected. A PPI network was constructed to validate the differential expression of KRT19 in combination with biosignature analysis, and the protein interaction between KRT19 and ESR1 was verified using STRING.

**Results:**

Ualcan showed that the expression of ESR1 and KRT19 was higher in THCA tissues than in normal thyroid tissues. E2 activation of ERα promoted the growth of PTC cells and tissues. si-KRT19 inhibited the proliferation, migration and invasion of PTC cells. KRT19 together with ERα formed the ERα/KRT19 signaling axis. E2 activation of the ERα/KRT19 signaling axis promoted the proliferation, migration, and invasion of PTC cells. ONT sequencing and STRING website verified that KRT19 is significantly differentially expressed in PTC and that ESR1 and KRT19 have protein interactions and are related to the estrogen signaling pathway.

**Conclusions:**

Using public databases, RNA sequencing, and bioinformatics, we discovered that E2 stimulates the ERα/KRT19 signaling axis to stimulate PTC proliferation, migration, and invasion.

**Supplementary Information:**

The online version contains supplementary material available at 10.1007/s40618-024-02473-5.

## Background

Thyroid cancer (THCA) is the most common malignancy in the endocrine system and is expected to become the fourth most common cancer by 2030 [1]. Papillary thyroid carcinoma (PTC) is the most common pathologic type of THCA, accounting for approximately 80% of all THCA [2]. PTC can spread to regional lymph nodes and compress adjacent organs in the early stage, which seriously affects the quality of life of patients [3]. Therefore, the search for molecular markers for the reduction of PTC incidence and metastasis rate is an urgent and critical task.

The epidemiological surveys have found significant gender and age differences in the rise in PTC incidence. The incidence of PTC in women is three times higher than in men, with a higher incidence in women of reproductive age, peaking between the ages of 40 and 49, and decreasing in incidence after menopaus [4, 5]. It has been reported that the use of oral contraceptives leads to an increased risk of cancer, as well as an increased risk of cancer in women using estrogen for gynecological diseases, but there is no change in the risk of cancer in postmenopausal women using low-dose estrogen replacement therapy [6, 7]. This body of experimental evidence suggests that high estrogen levels in vivo may get involved in promoting PTC development. Classical estrogen signaling pathway are mediated by estrogen receptors (ERs), members of the large family of nuclear transcription factors [8]. The estrogen receptor α (ERα) and β (ERβ) are two different ERs encoded by the ESR1 and ESR2, respectively [9]. E2, Erα and ERβ have also been shown to be involved in the pathogenesis of PTC [10, 11]. Up-regulation of ERα expression promotes the development of PTC, while ERβ plays a protective role in PTC by regulating anti-proliferative and pro-apoptotic signals [12–14]. However, the molecular mechanism by which estrogen/ERα promotes PTC growth and metastasis is still under investigation.

It has been reported in the literature that keratin 19 (KRT19), a protein-coding gene with a molecular weight of 40–44 kDa, encodes a family of keratin proteins that are intermediate filament proteins responsible for the structural integrity of epithelial cells [15]. It has been discovered that KRT19 is highly expressed in a number of cancers, including lung adenocarcinoma, ovarian cancer, and breast cancer; and it promotes the growth and metastasis of these cancers and is thought to have some bearing on the prognosis of these cancer [16–19]. Similarly, dysregulated expression of KRT19 in malignant thyroid lesions is thought to be useful in evaluating indeterminate thyroid nodules [20, 21]. In recent years, KRT19 has also been found to have a beneficial role in the diagnosis of PTC [22–24]. KRT19 and ERα play a promoting role in a variety of cancers, but there are few reports on the relationship between the two effects. It has been documented that KRT19 expression is downregulated when ERα is knocked down in breast cancer [25]. However, the role of KRT19 with PTC estrogen is still unclear.

In this study, we used clinical Oxford Nanopore Technologies (ONT) sequencing and gene expression information from THCA patients in the TCGA database to investigate changes in KRT19 in THCA and neighboring non-cancerous tissues. In order to identify potential targets for the future reduction of PTC incidence and metastasis, this study sought to uncover the potential molecular mechanism of the correlation between KRT19 expression and PTC estrogen as well as the molecular mechanism of KRT19 in PTC.

## Methods

### Ualcan database search

The UALCAN database (http://ualcan.path.uab.edu/) combines clinical data from 31 cancer types with TCGA tertiary RNA-seq to facilitate easy access to publicly available canceromics data (TCGA and MET 500); it also allows for the electronic validation of potential genes of interest or biomarker identification; and it offers features like gene expression graphs and patient survival data based on gene expression [26]. Methods: After entering the website, the Analysis option was selected and the Scan by Genes search box was used. Search criteria: ① Enter Gene symbol: ESR1, KRT19; ② TCGA dataset: Thyroid carcinoma; ③ Explore.

### Cell culture and treatment

The human papillary thyroid carcinoma cell lines TPC-1 (Catalog No. CL-0643) and KTC-1 (Catalog No. CL-0649) were purchased from Wuhan Procell Life Science Co., Ltd. The European Collection of Animal Cell Cultures provided the human thyroid follicular epithelial cell Nthy-ori 3 − 1 (ECACC Catalogue No. 90011609). STR validation was carried out on the cell lines utilized in the research in order to rule out cross-contamination of the lines. TPC-1, KTC-1, and Nthy ori 3 − 1 were cultured in RPMI-1640 (Gibco) basal medium containing 10% fetal bovine serum (FBS, Procell) and 1% penicillin-streptomycin (Procell). The cell culture environment was 37 ℃ and 5% CO_2_. β-estradiol(E2, #E8140)and ERα receptor antagonist, MPP ( Catalog No. C3089) were purchased from Solarbio and APExBIO, respectively. Both E2 and MPP were dissolved in DMSO at storage concentrations of 20 μm and 5 μm and sequentially stored at 4℃ and − 20℃. Concentrations of 10nM E2 and 20 μm MPP were used.

### Xenograft model

**Fig. 1:**
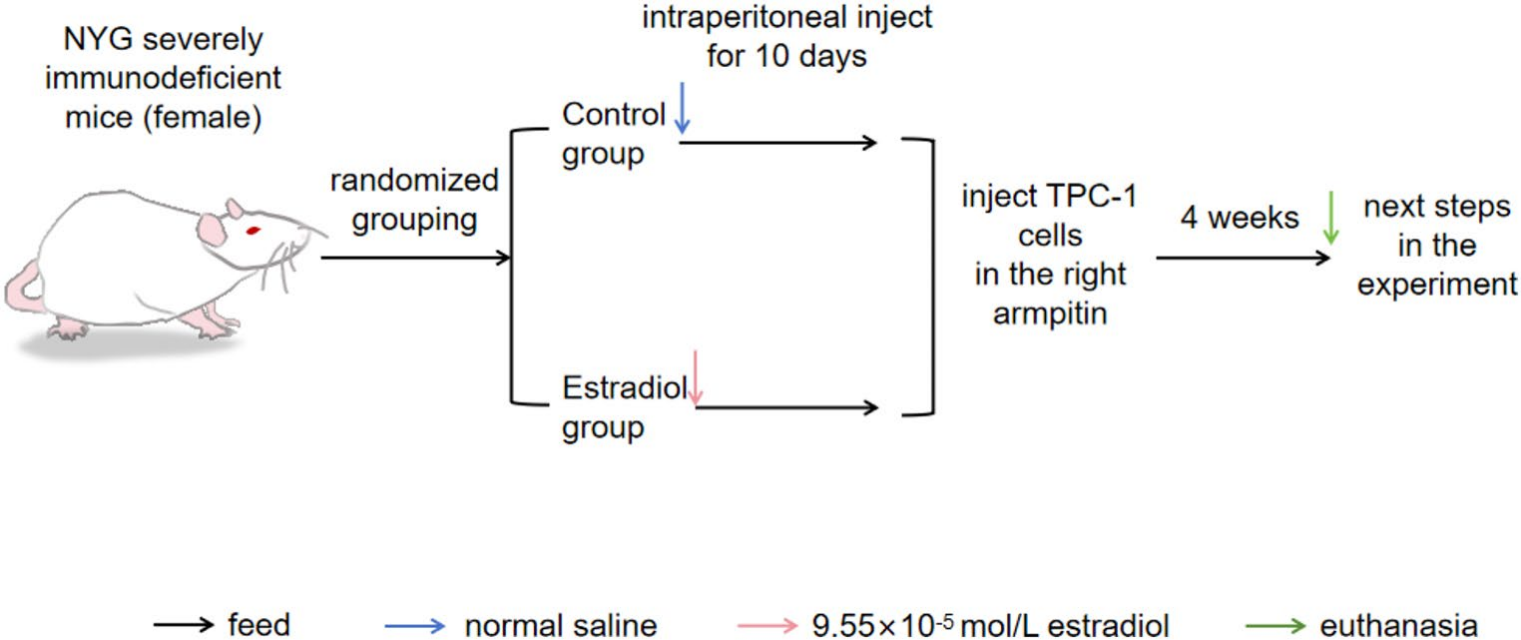
The flow chart of the animal experiment

We bought ten female NYG severely immunodeficient model mice from Liaoning Changsheng Biological Co., Ltd. when they were between 4 and 8 weeks old. After 1 week of adaptive feeding, animals were randomized to groups of 5 animals each. The control group received an intraperitoneal injection of normal saline, while the experimental group’s mice received an injection of E2 (9.55 × 10^− 5^mol/L, #E8140)dissolved in saline. Mice were injected subcutaneously into the right axilla with 1 × 10^7^ TPC-1 cells. Tumor size was measured and recorded at an average interval of 3 days from day 7 after cell injection. About 4 weeks after cell inoculation, mice were euthanized, tumors were surgically removed, photographed, weighed and tumor tissues were collected for the next experimental analysis. (Tumor volume = (length × width^2^)/2). The flow chart of the animal experiment is shown in Fig. 1. The Laboratory Animal Welfare and Ethics Committee of Jinzhou Medical University approved all animal procedures (241074).

### Immunohistochemical staining

The tissues were preserved in paraformaldehyde (4%). 5 μm was the slice thickness. Sections were routinely deparaffinized and rehydrated. After 30 min of cooling at room temperature, the antigens were recovered under high pressure using 0.01 mol/L sodium citrate buffer (pH = 6.0). Immunohistochemical reactions were performed according to Mouse High Sensitivity Two Step Immunohistochemical Detection Kit instructions(ZSGB-BIO, PV-9005). Slice samples were reacted with ERα antibody (#AF6058, Affinity) and Ki-67 antibody (#ab279653, Abcam) for an entire night at 4 °C. Incubate for 5 min at room temperature according to the instructions of the DAB Color Development Kit (ZSGB-BIO, ZLI-9018). Rinse in tap water, stain with hematoxylin (Solarbio) and differentiate with 1% ethanol hydrochloride. Ethanol gradient dehydration, xylene clarification and neutral gum mounting. The immunohistochemical staining was scored using the staining intensity and the percentage of positive cells. The intensity of staining was scored on the following four-point scale: 0 (no staining), 1 (weak staining), 2 (moderate staining), and 3 (strong staining). The experiment was repeated five times.

### Western blot

Total cellular proteins were extracted with RIPA lysis solution (Solarbio) on ice for cell experiments. For animal experiments, tumor tissues were soaked in an appropriate amount of RIPA lysis solution, tissues were minced by ophthalmic clipping, and tissue pellets were broken using ultrasound. Centrifuge at 12,000rmp for 25 min at 4℃,discard the pellet, take the supernatant and determine the extracted protein concentration by BCA. Samples were added to 5×loading buffer and then boiled for 5 min at 100 °C. Separated by SDS-PAGE electrophoresis. Subsequently, transferred proteins to PVDF and closed with TBST containing 1% BSA for 2 h. The PVDF were sectioned according to their molecular weight. Following overnight incubation at 4 °C with primary antibodies, TBST was washed, and then a 2-hour room temperature incubation period was allowed for the secondary antibodies coupled with horseradish peroxidase (HRP). Optical density measured by ChemiDocTM Imaging System gel imager(#733BR2213, BIO-RAD). Image J V1.8.0 software (National Institutes of Health, Bethesda, MD, USA) was used to analyze signal intensities. Primary antibodies used in this paper include: anti-ERα (#AF6058, Affinity);anti-KRT19 (#ab7755, Abcam); anti-PCNA (#WL03213, wanleibio); anti-E-cadherin (#AF0131, Affinity); anti-Vimentin(#AF7013, Affinity) and anti-GAPDH (#ab9485, Abcam). HRP-conjugated secondary antibodies, including: HRP Goat Anti-Rabbit IgG (H + L) Antibody (Catalog No. K1223, APExBIO)and HRP Goat Anti-Mouse IgG (H + L) Antibody (Catalog No. K1221, APExBIO).

### 5-Ethynyl-2′-deoxyuridine (EdU) labeling

Proliferation capacity of TPC-1 and KTC-1 was determined following transfection of cells in 6-well plates or treatment with E2 and MPP according to BeyoClick™ Edu-594 Cell Proliferation Assay Kit (Beyotime) instructions. Fix with 4% paraformaldehyde. Washing with 3% BSA in PBS. The Click Reaction Solution was made in accordance with the directions and let to sit at room temperature in the dark for 30 min. Nuclei were stained with Hoechst 33,342 (1:1000) provided with the PBS dilution kit for 10 min at room temperature in the dark. The fluorescence intensity of cells was observed under a fluorescence microscope.

### Transient KRT19 knockdown

Both the KRT19 siRNA and the negative control siRNA were purchased from GenePharma (www.genepharma.com). Using Lipofectamine™ 2000 Transfection Reagent (Invitrogen) and the transfection method recommended by the manufacturer, these siRNAs were transfected into PTC cells. Cell samples were collected 2 days after transfection. Detect knockdown efficiency using Western blot.

### Cell counting kit-8(CCK-8)

Following transfection, cells were collected and seeded in 96-well plates at 5 × 10^3^ cells/well, and CCK-8 assays were performed daily for the next three days to determine cell proliferation. Five replicate wells were set for each group. In summary, each 96-well was filled with 10uL of CCK-8 solution (Catalog No. K1018, APExBIO), and the incubation process was allowed to continue for 4 h in the cell incubator. For every well, the optical density was measured at 450 nm. There were three iterations of the experiment.

### Immunofluorescence staining

Following transfection, digested cells were gathered and planted to 24-well plates, where they were left overnight. Fixation was performed with 4% paraformaldehyde for 30 min. For thirty minutes, blocking was done in PBS containing 3% BSA. Lastly, let the primary antibodies incubate for an entire night at 4 °C. Incubate for 2 h at room temperature in the dark with fluorescent secondary antibodies of the corresponding species. Finally, nuclei were stained with DAPI (Solarbio) solution for 10 min at room temperature in the dark. Finally, fluorescence intensity was observed with a microscope. Primary antibodies include, anti-ERα(#AF6058, Affinity); anti-ki-67(#AF0198, Affinity); Fluorescent secondary antibodies include, FITC AffiniPure Goat Anti-Rabbit IgG(H + L)(Catalog NO.E031220-01, EARTHOX).

### Scratch test

After transfection, seed 1 × 10^6^ cells/well into 6-well plates. Then, using a pipette tip, the cell monolayers were scraped. Rinse floating cells with serum-free medium. After scratching, each group was photographed at 0, 24, and 48 h. The experiment was repeated three times.

### Transwell

Experimental measurements of cell migration and invasion were performed using 24-well transwell inserts (8.0 μm pore size, Corning, NY, USA) plates. After Matrigel (Catalog NO.356234, Corning) was diluted 1:15 with serum-free medium, 100µL of diluted Matrigel was added to the upper chamber surface of the insert, and the plate was incubated and allowed to solidify. One hundred microliters of cell suspension (3 × 10^4^ cells/100uL for migration assay; 5 × 10^4^ cells/100uL for invasion assay) into the upper chamber and 600uL of cell complete cell medium with 10% FBS into the lower chamber. Incubated for 24 h. Remove the cell and gel from the upper chamber. Paraformaldehyde (4%), fixed, and Crystal Violet Ammonium staining. Dried and then photographed under a microscope. To determine cell migration, the steps are identical. However, Matrigel is not added. The experiment was repeated three times.

### RNA sequencing

We collected 15 PTC patients who were diagnosed at the Department of Thyroid Surgery of the First Affiliated Hospital of Jinzhou Medical University from September 2021 to May 2022, took fresh cancer and adjacent non-cancerous tissue pairs, cryopreserved in liquid nitrogen, and sent them to Biomarker Biotechnologies Co.Ltd.92 (Beijing, China) for RNA sequencing. Following the manufacturer’s instructions, total RNA was extracted using an RNA extraction kit (Takara Bio Inc., Kyoto, Japan). The mRNA fraction of total RNA was assessed using an Implen NanoPhotometer spectrophotometer (Implen, Westlake Village, CA, USA). RNA was reverse transcribed into cDNA and cDNA libraries were built with a cDNA-PCR sequencing kit (SQK-PCS109; ONT, Oxford, UK). Fourteen cycles of PCR amplification were performed using LongwellTag DNA polymerase (New England Biolabs, Ipswich, MA, USA).Final cDNA libraries were analyzed using FLO-MIN109 flow cells (R9 Version, FLO-PRO002; ONT) and the PromethION 48 platform (ONT). GffCompare (version 0.9.8) was used to compare with transcripts from the genome to identify novel genes and transcripts. Prior to enrollment, informed permission was acquired from every eligible patient. The study started after obtaining approval from the Ethics Committee of The Third Affiliated Hospital of Jinzhou Medical University(Approval Number: KX2021011).

### Screening of differentially expressed genes (DEGs)

Fold change (FC) of gene expression was compared between cancer tissues and adjacent non-cancerous tissues. Differential expression was analyzed using edgeR software (version 3.8.6) with | log 2 Fold change (FC) | ≥ 1.5 and *p* < 0.05 as thresholds for screening significant DEGs.

### Protein-protein interaction (PPI) analysis

PPI networks of the top 500 DEGs screened based on RNA sequencing were constructed with the STRING (https://string-db.org/). In addition, the website can import the obtained genetic data into Cytoscape (version 3.9.1). Molecular Complex Detection (MCODE) was performed to filter the largest PPI network module with a degree cutoff = 2, node fraction cutoff = 0.2, and k-core = 2 [27, 28].

### Gene ontology (GO) and Kyoto Encyclopedia of genes and genomes (KEGG) enrichment analysis

Based on the GO database, the selected DEGs were functionally enriched from three aspects: cellular component, biological process and molecular function. The KEGG analysis method was used to analyze the metabolic pathways and signal transduction pathways involved in DEGs, and significance of gene enrichment in each pathway was calculated. DEGs were subjected to GO functional analysis as described above using the DAVID (https://david.ncifcrf.gov/)online database [29].

### Statistical analysis

All experimental data in this study are presented as mean ± standard deviation (SD). Unpaired t-tests, one-way analysis of variance (ANOVA) for comparisons between multiple groups, and Tukey’s multiple comparison tests were performed using GraphPad Prism 9 software to test for statistical differences between groups. Statistical significance was expressed as *P < 0.05, **P < 0.01, ***P < 0.001, respectively.

## Results

### Relationship between ESR1, KRT19 mRNA expression and THCA

**Fig. 2:**
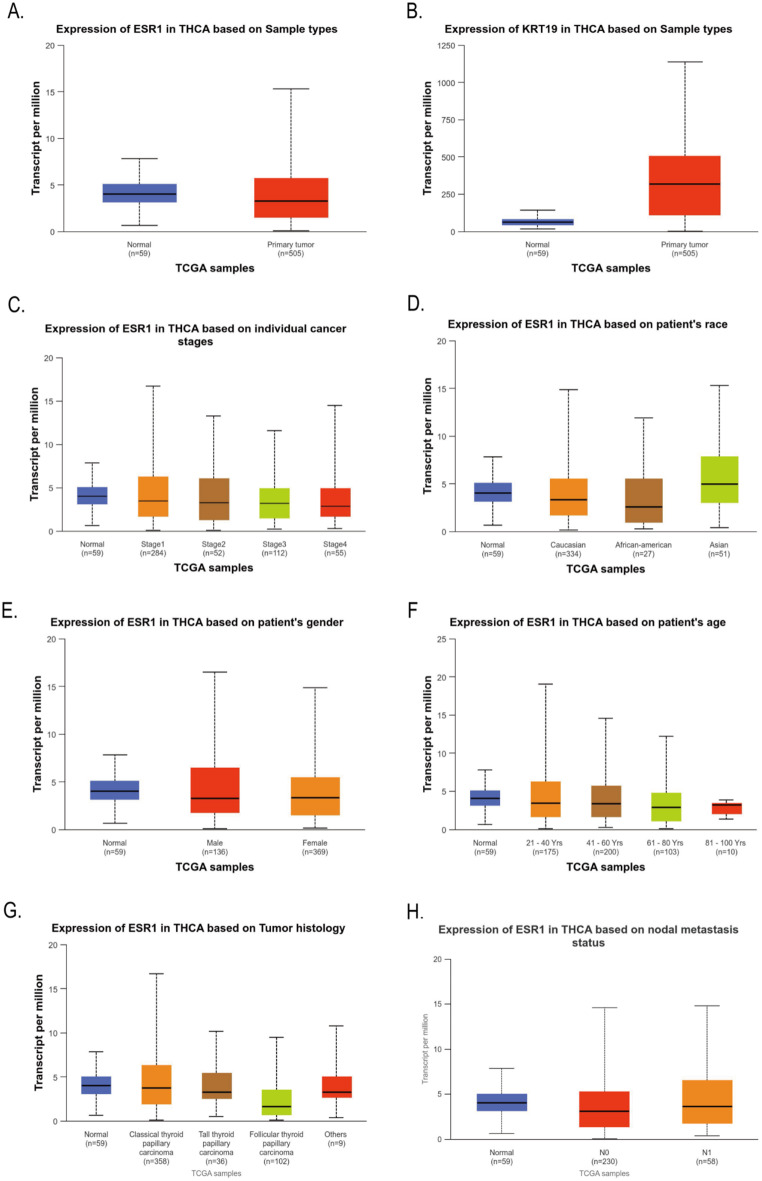
Ualcan analysis of ESR1 and KRT19 mRNA expression in THCA tissues from the TCGA database. (**A**) ESR1 expression varies between THCA and normal tissues; (**B**) Differential expression of KRT19 in THCA tissues and normal tissues; **C**-**H**. Differential expression of ESR1 in THCA tissues with different cancer stages, races, genders, age groups, histological types and lymph node metastasis. All *P* < 0.05.

**Fig. 3:**
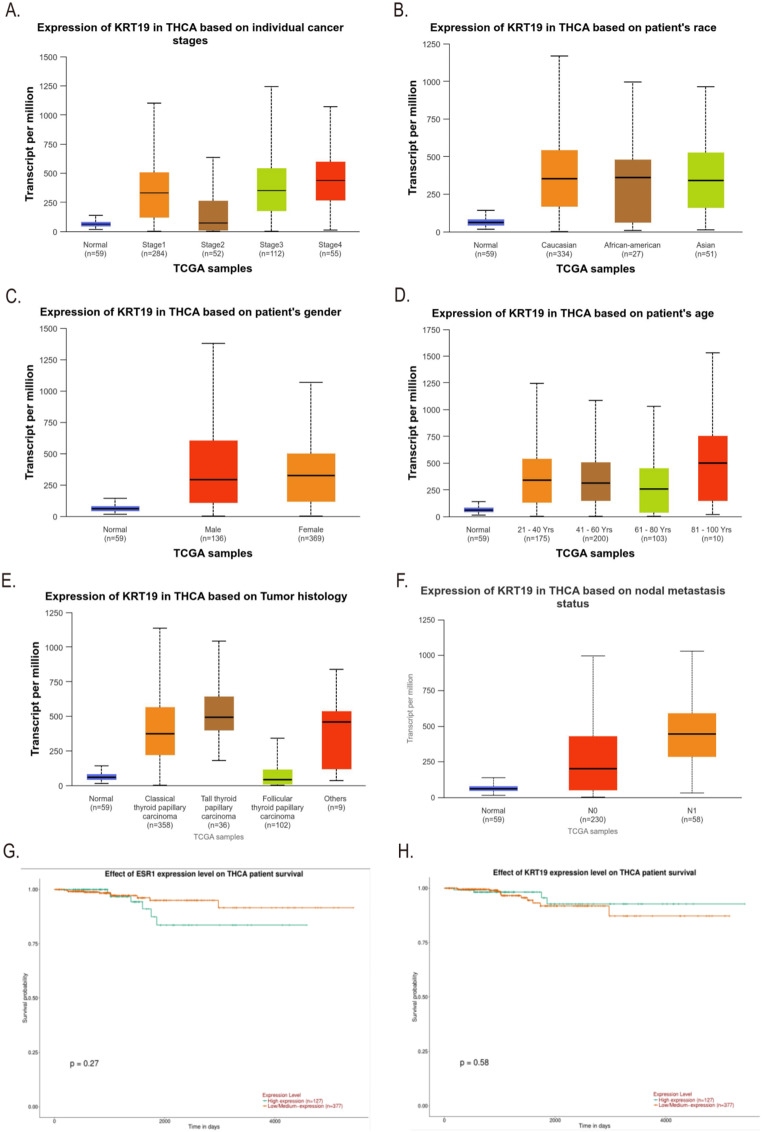
Relationship between KRT19 mRNA expression and THCA in the TCGA database and survival analysis of ESR1, KRT19 and THCA. **A**-**F**. ESR1 was differentially expressed in THCA tissues at different cancer stages, different races, genders, age groups, histological subtypes and nodular metastasis status; all *P* < 0.05. Using Ualcan online analysis, **G**. the effect of different expression levels of ESR1 on the survival of THCA patients; **H**. the effect of different expression levels of KRT19 on the survival of THCA patients. All *P* > 0.05.

The differential expression of ESR1 and KRT19 in 59 normal tissues and 505 THCA tissues in the TCGA database was analyzed by Ualcan. Compared to normal thyroid tissues, THCA tissues exhibited greater levels of ESR1 (*P* = 2.6555E-07, Fig. 2A); also, THCA tissues expressed KRT19 more than normal thyroid tissues did (*P* = 1.6244E-12, Fig. 2B). We also found by Ualcan analysis of the TCGA database that the expression of ESR1 and KRT19 in different cancer stages, races, genders, age groups, histological subtypes, and lymph node metastasis status were significantly different in THCA (All *P* < 0.05, Figs. 2C - 3F). In addition, we investigated the correlation between differential expression of ESR1, KRT19 mRNA and THCA prognosis. Ualcan online was used to analyze the differential expression of ESR1 and KRT19 for survival. The low and high expression of ESR1 and KRT19 were 377 and 127 cases, respectively. The differential expression of ESR1 and KRT19 had no statistical difference in the survival of THCA patients (*P* = 0.27, *P* = 0.58, Fig. 3G-H).

### Estrogen promotes the growth of PTC xenografts in mice

**Fig. 4:**
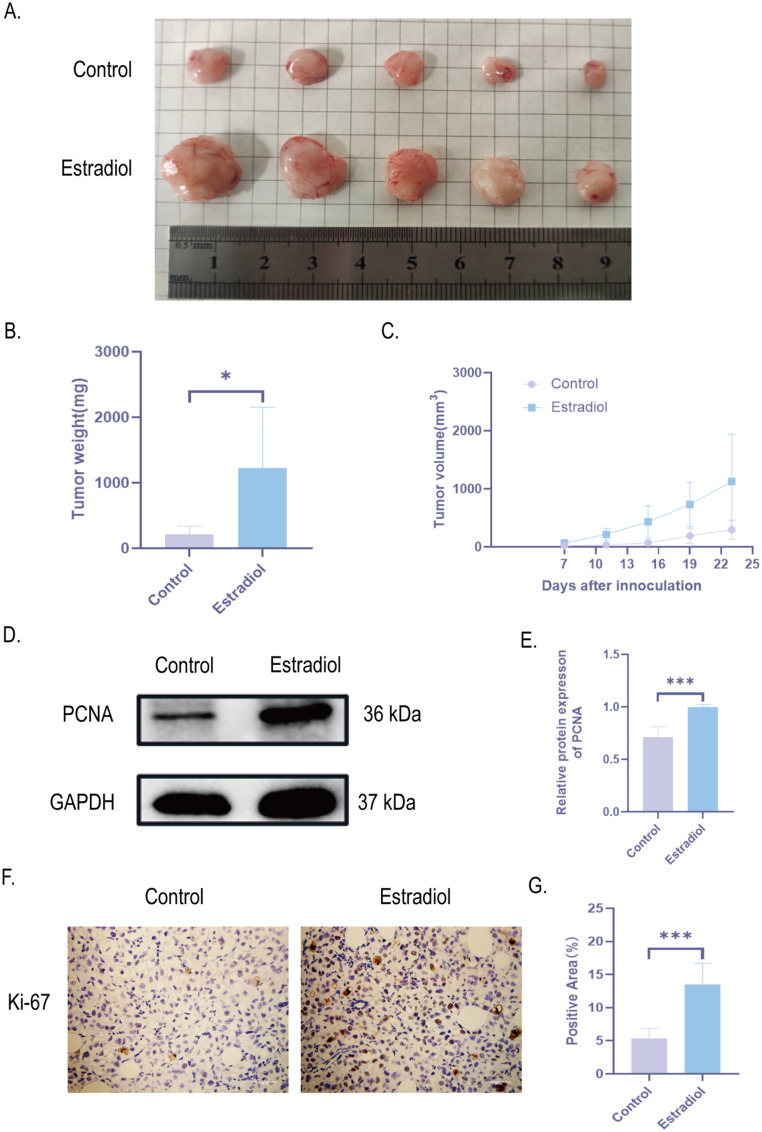
Estrogen promotes growth of PTC xenografts in mice. **A**-**C**. Effect of E2 on the external characteristics, weight and volume of PTC xenograft tissues; **D**, **E**.Weaster Blot was used to determine PCNA protein expression levels and quantitative maps in mouse PTC xenograft tissues. Band intensities were normalized to GAPDH. **F**, **G**. Immunohistochemical staining and quantification plots of Ki-67 expression in tumors. All data are presented as mean ± SD. *p < 0.05, **p < 0.01, ***p < 0.001. PCNA, Ki-67, a nuclear antigen of cell proliferation activity

To validate the effect of estrogen on PTC growth, we constructed a PTC xenograft model. We simulated the basic condition of higher estrogen levels in women of reproductive age and injected E2 intraperitoneally into mice. The control group injected with saline. 1 × 10^7^ TPC-1 were injected subcutaneously into the right axilla of both groups. It was found that tumor volume and tumor weight in E2 group were higher than those in control group (Fig. 4A-C). In addition, we further verified that the expression of PCNA and Ki-67 was significantly increased in the E2 group compared with PTC tissues in the control group by detecting the proliferation factor PCNA by Western blot and Ki-67 by immunohistochemistry, indicating that E2 could increase the proliferation level of PTC tissues (Fig. 4D-G). In summary, estrogen can promote the growth of PTC xenograft tissue.

### E2 promotes the development of PTC through activating ERα

**Fig. 5:**
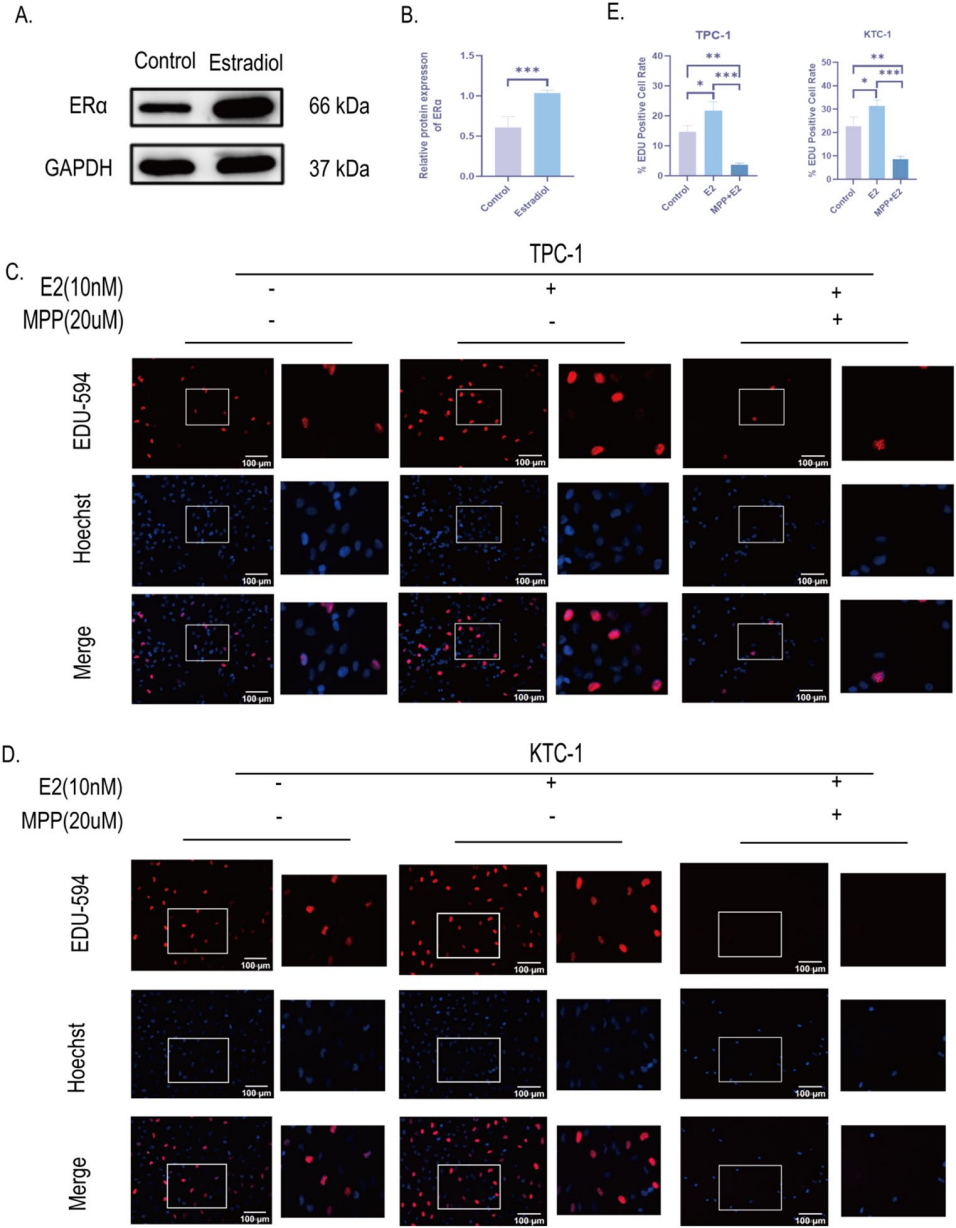
E2 promotes PTC proliferation by activating ERα. **A**, **B**. Weaster Blot to determine ERα protein expression levels and quantification profiles in PTC xenograft tissues. Band intensities were normalized to GAPDH. **C**-**E**. Fluorescence plot (x200) and quantification plot of DNA replication activity detected by EDU-594 cell staining assay in TPC-1 and KTC-1 cells treated with 20 μm MPP and 10 nM E2. The right panel in the fluorogram represents the locally enlarged fluorogram at the same location. Fluorescence and quantification plots of TPC-1 and KTC-1 DNA replication activity detected by the EDU-594 cell staining assay. Cell nuclei were stained with Hoechst 33,342 (blue). Scale bar = 100 μm. All data are presented as mean ± SD. *p < 0.05, **p < 0.01, ***p < 0.001

To verify the mechanism of E2 promoting PTC growth, we further examined ERα expression in PTC xenograft tissues by Western blot. The results showed that ERα protein expression was significantly increased in E2 group compared with control group (Fig. 5A-B). This suggests that E2 promotes PTC growth through activation of ERα in PTC tissues. Next, we treated two human PTC cells (TPC-1 and KTC-1) in vitro with E2 and MPP, an ERα-specific receptor antagonist. Activation of ERα by E2 significantly promoted DNA replication activity and promoted cell proliferation in PTC cells as detected by EDU-594 cell proliferation assay; conversely, inhibition of ERα by MPP also inhibited the proliferation level of PTC cells (Fig. 5C-E). These results indicate that E2 promotes PTC development by activating ERα.

### Knockdown of KRT19 inhibits cell proliferation, migration and invasion of PTC

**Fig. 6:**
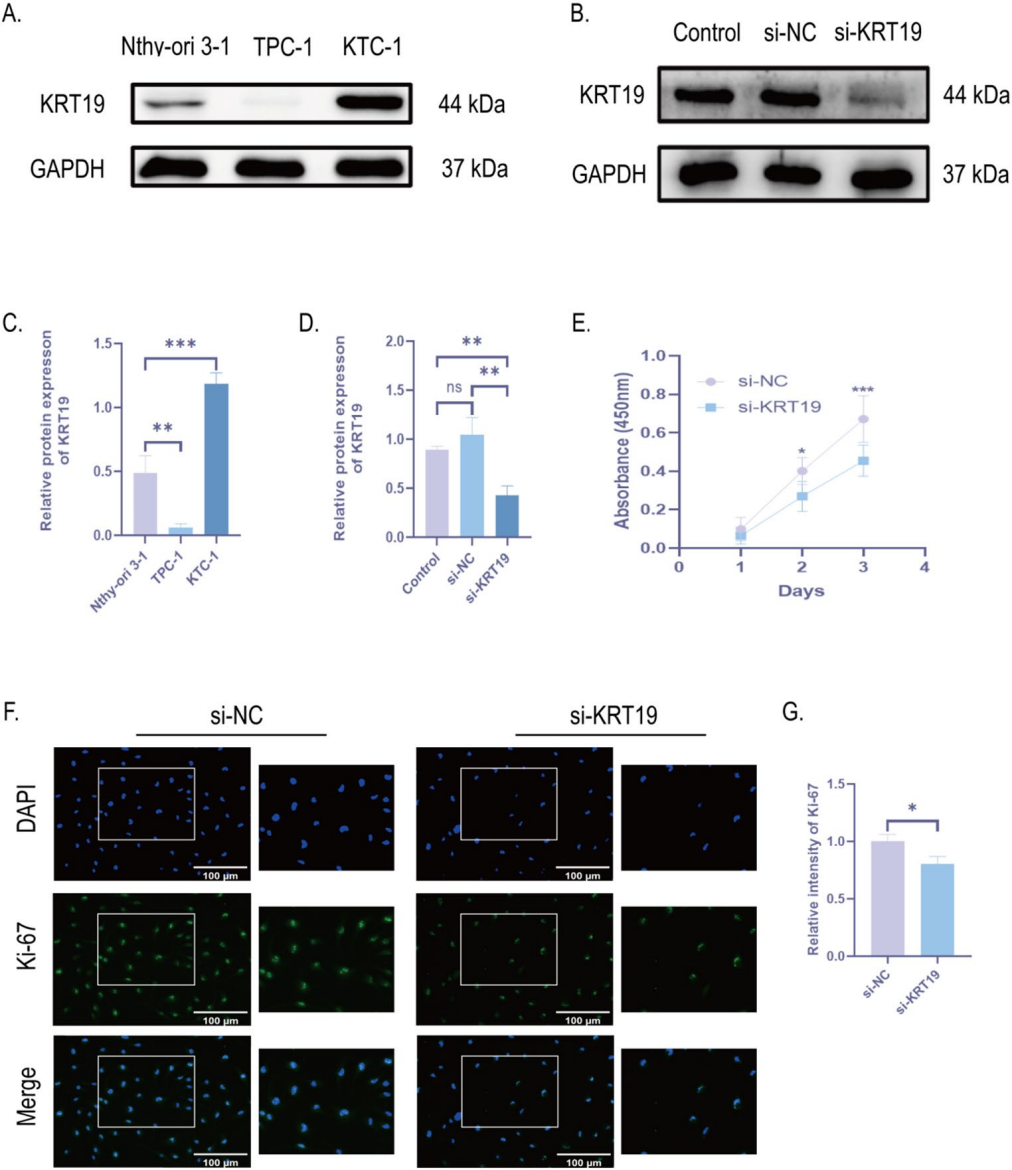
Knockdown of KRT19 inhibits cell proliferation of PTC. **A**, **C**. Human thyroid follicular epithelial cells (Nthy-ori 3 − 1) and human PTC cell lines (TPC-1 and KTC-1) utilize Western blot to assess the protein expression and quantification of KRT19. **B**, **D**. Western blot detect the knockdown rate of blank, negative control (denoted as si-NC) and knockdown KRT19 groups (denoted as si-KRT19) in KTC-1. Band intensities were normalized to GAPDH. **E**. CCK-8 detected cell growth in KTC-1 after si-KRT19. **F**-**G**. Immunofluorescence profile (x200) and quantification profile of Ki-67 in KTC-1 following si-KRT19. The right panel in the fluorogram represents the locally enlarged fluorogram at the same location. Scale bar represents 100 μm. All data are presented as mean ± SD. *p < 0.05, **p < 0.01, ***p < 0.001. ns, the difference was not significant

**Fig. 7:**
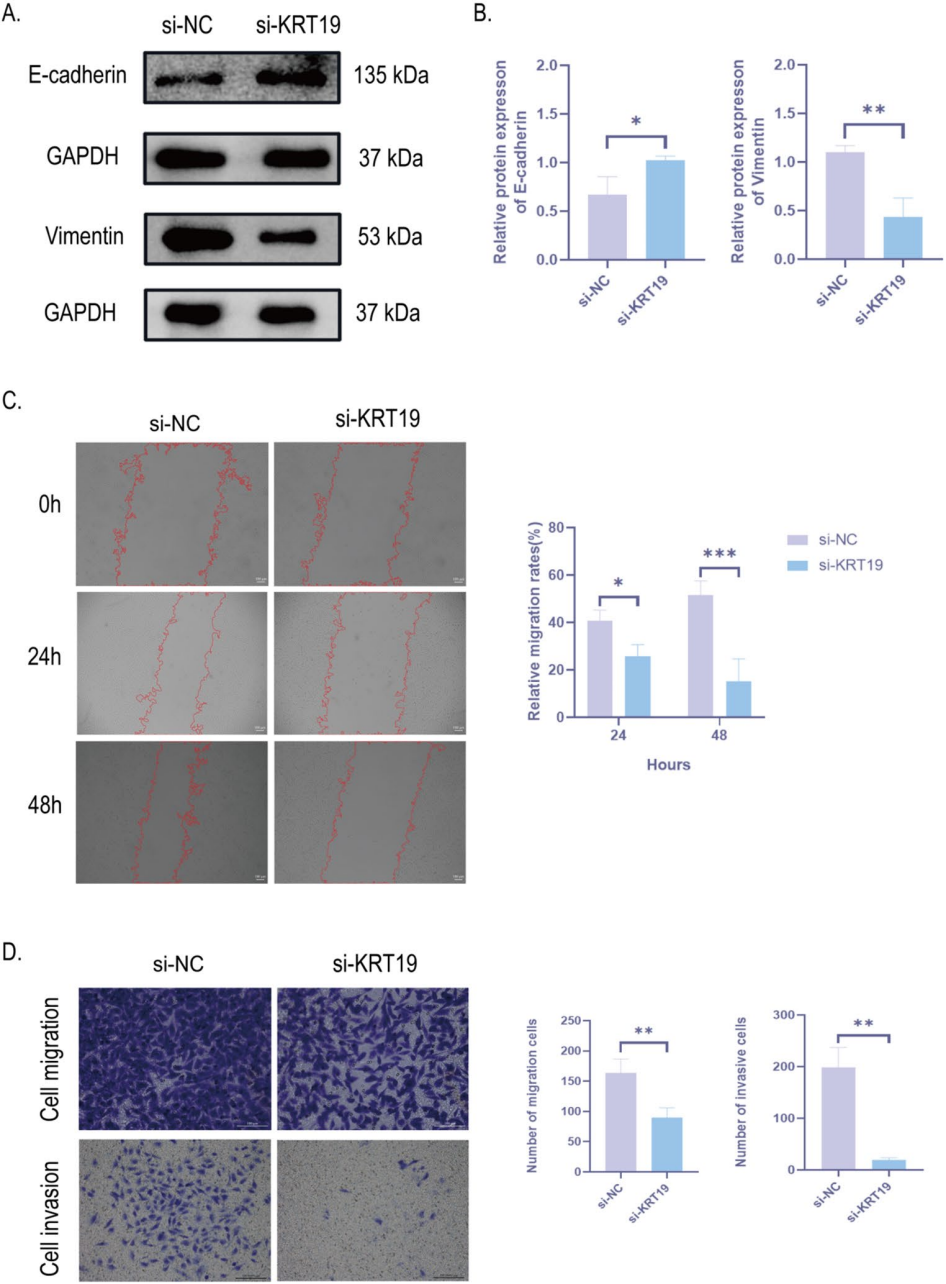
Knockdown of KRT19 inhibits the migration and invasion of PTC cells. **A**, **B**. Protein expression and quantification plots of E-cadherin and vimentin in si-NC and si-KRT19 groups of KTC-1 cells detected by Western blot. Band intensities were normalized to GAPDH. **C**. Migration and quantification plot of KTC-1 after si-KRT19 detected by cell scratch. **D**. Migration and invasiveness of KTC-1 after si-KRT19 detection by transwell and quantification plot. Scale bar = 100 μm. All data are presented as mean ± SD. *p < 0.05, **p < 0.01, ***p < 0.001

Next, we further investigated how KRT19 affects PTC biofunction. First, we examined the expression of KRT19 in human thyroid follicular epithelial cells (Nthy-ori 3 − 1) and two human papillary thyroid carcinoma cells (TPC-1 and KTC-1) by Western Blot and further verified that KRT19 expression was significantly up-regulated in human PTC cells. Interestingly, the TPC-1 cell line did not show a significant up-regulation of KRT19, which may be related to the fact that the cells did not express KRT19, so subsequent experiments were validated with the KTC-1 cell line (Fig. 6A, C). Next, to test how KRT19 affects PTC development, we knocked down KRT19 in KTC-1 with small interfering RNA (si-KRT19), scrambled siRNA was used as the control group (si-NC), and the blank group (control expression) was used to rule out the effect of transfection reagents in the cells. The final gene knockdown rate was confirmed with Western Blot (Fig. 6B, D). The proliferation of PTC cells was then verified by the CCK8 assay and Ki-67 immunofluorescence labeling. The findings demonstrated that, in comparison to the si-NC group, PTC cell proliferation was reduced by KRT19 knockdown (Fig. 6E-G). In addition, we also detected the expression levels of EMT progression markers (E-cadherin and vimentin) by Western blot, and the results showed that si-KRT19 significantly increased the expression of E-cadherin and inhibited the protein expression level of vimentin (Fig. 7A-B). Cell scratch and transwell assays were further applied to verify migration and invasiveness of PTC cells. si-KRT19 significantly inhibited the migration and invasion ability of KTC-1 cells (Fig. 7C-D). In summary, KRT19 can promote the migration, invasion, and EMT process of PTC cells.

### ERα is related to KRT19 and constitutes the ERα/KRT19 signaling axis regulated by E2

**Fig. 8:**
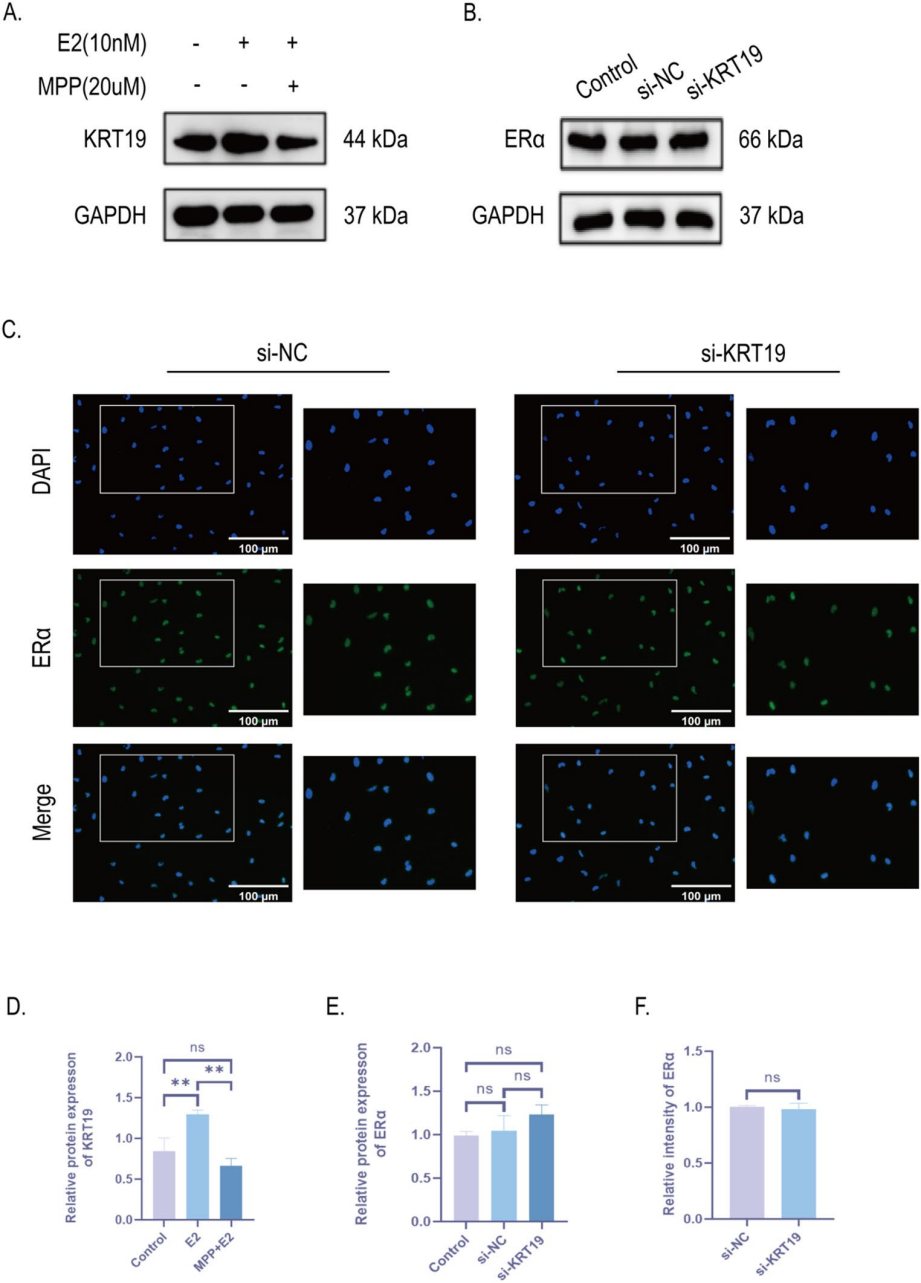
ERα interacts with KRT19 and constitutes the ERα/KRT19 signaling axis regulated by E2. **A**, **D**. KRT19 protein expression and quantification were measured by Western blot in two groups of cells treated with 20 μm MPP and 10nM E2. **B**, **E**. Western blot detection of ERα protein expression and quantification plots of the blank, si-NC, and si-KRT19 groups in KTC-1. Intensities were adjusted in relation to GAPDH. **C**, **F**. Immunofluorescence profile (x200) and quantification profile of ERα in KTC-1 after si-KRT19. The right panel in the fluorogram represents the locally enlarged fluorogram at the same location. Scale bar represents 100 μm. All data are presented as mean ± SD. **p < 0.01. ns, the difference was not significant

To validate the relationship between ERα and KRT19, we administered E2 and MPP, respectively, to KTC-1 cells. KRT19 protein expression levels were found to be elevated after E2 stimulation by Western Blot, which is consistent with our previous trend of ERα activation with the same intervention (Fig. 8A, D). Therefore, we suggest an interaction relationship between ERα and KRT19. Interestingly, however, we showed no significant statistical difference in the decrease of KRT19 after administration of MPP to inhibit ERα, although there was a trend towards down-regulation compared with the blank group (Fig. 8A, D). We think this might have to do with the fact that E2 can activate other ERs in addition to ERα, which in turn influences KRT19 expression. Using Western Blot and immunofluorescence staining, we were able to confirm the unique interaction between ERα and KRT19 by seeing that there was no significant change in ERα expression following si-KRT19.(Fig. 8B-C, E, F). Based on these experimental findings, we deduced that E2 activates the ERα/KRT19 unidirectional signaling axis in PTC, which is made up of ERα and KRT19.

### E2 promotes the proliferation, migration and invasion of PTC cells by activating the ERα/KRT19 signaling axis

**Fig. 9:**
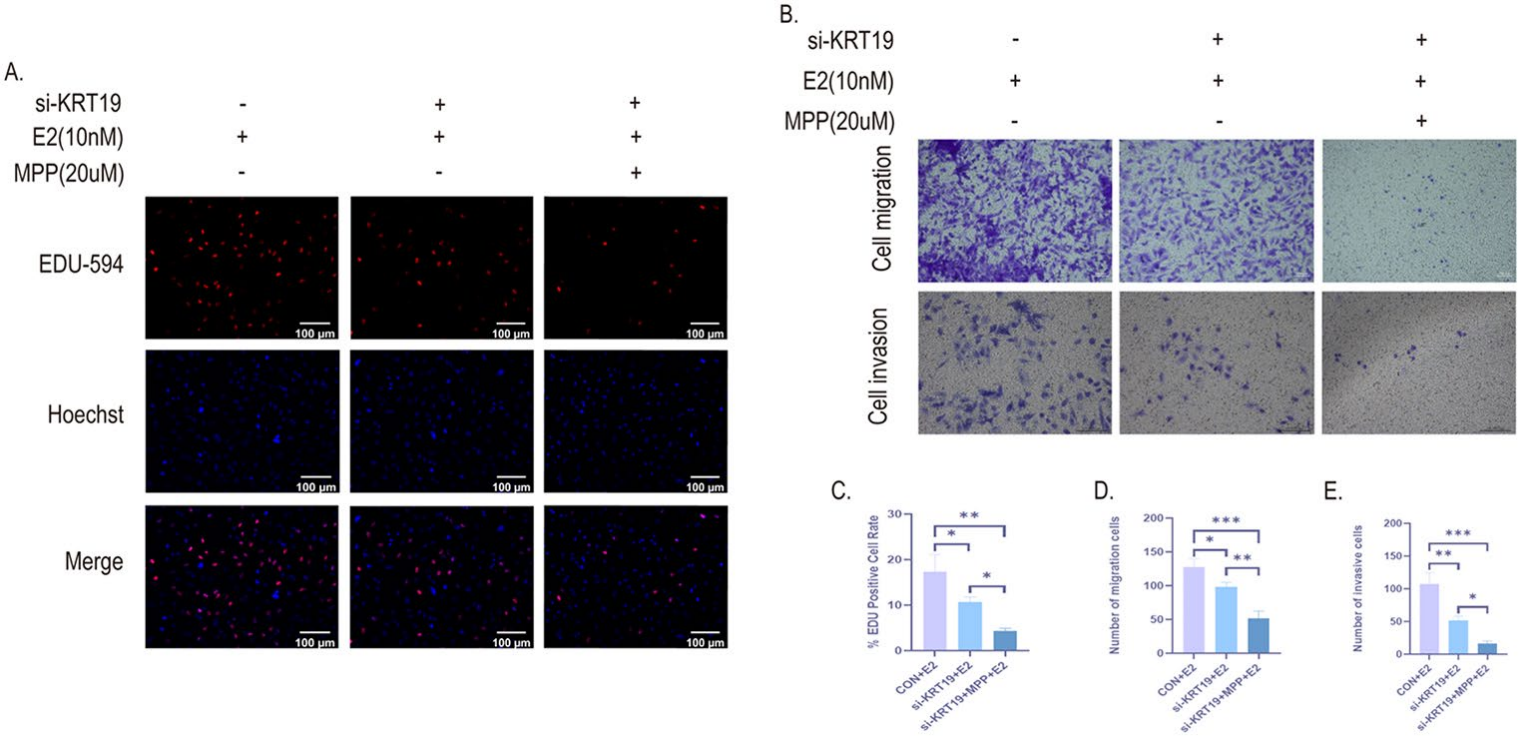
E2 promotes PTC proliferation, migration and invasion via activation of the ERα/KRT19 signaling axis. First, two groups of KTC-1 cells were treated with si-KRT19, followed by 20 μm MPP and 10nM E2 for 24 h. **A**, C. Fluorescence (x200) and quantification plots of DNA replication activity of three groups of KTC-1 cells treated differently by EDU-594 cell staining. **B**, **D**, **E**. The invasive and migrating characteristics of KTC-1 cells in each group were assessed using the Transwell Assay and quantitative plotting. Scale bar = 100 μm. All data are presented as mean ± SD. *p < 0.05, **p < 0.01, ***p < 0.001

To further investigate the role of ERα/KRT19 signaling axis in the development of PTC cells, we used EDU labeling to determine cell proliferation. The findings demonstrated that PTC cells’ DNA replication activity was reduced and their proliferation was inhibited following si-KRT19 (si-KRT19 + E2 expression) in comparison to the blank group (CON + E2 expression). However, their proliferation level remained higher than that of the entire signaling axis group that inhibited ERα/KRT19 (si-KRT19 + MPP + E2 expression) (Fig. 9A, C). Transwell determined PTC migration and invasion in each group. Similarly, cell migration and invasion of PTC following inhibition of si-KRT19 + MPP were significantly reduced (Fig. 9B, D-E). This suggests that E2 promotes PTC growth and metastasis via activating the ERα/KRT19 signaling axis.

### Screening of significantly upregulated differentially expressed genes (DEGs) in PTC tissues and adjacent non-cancerous tissues by ONT sequencing

**Fig. 10:**
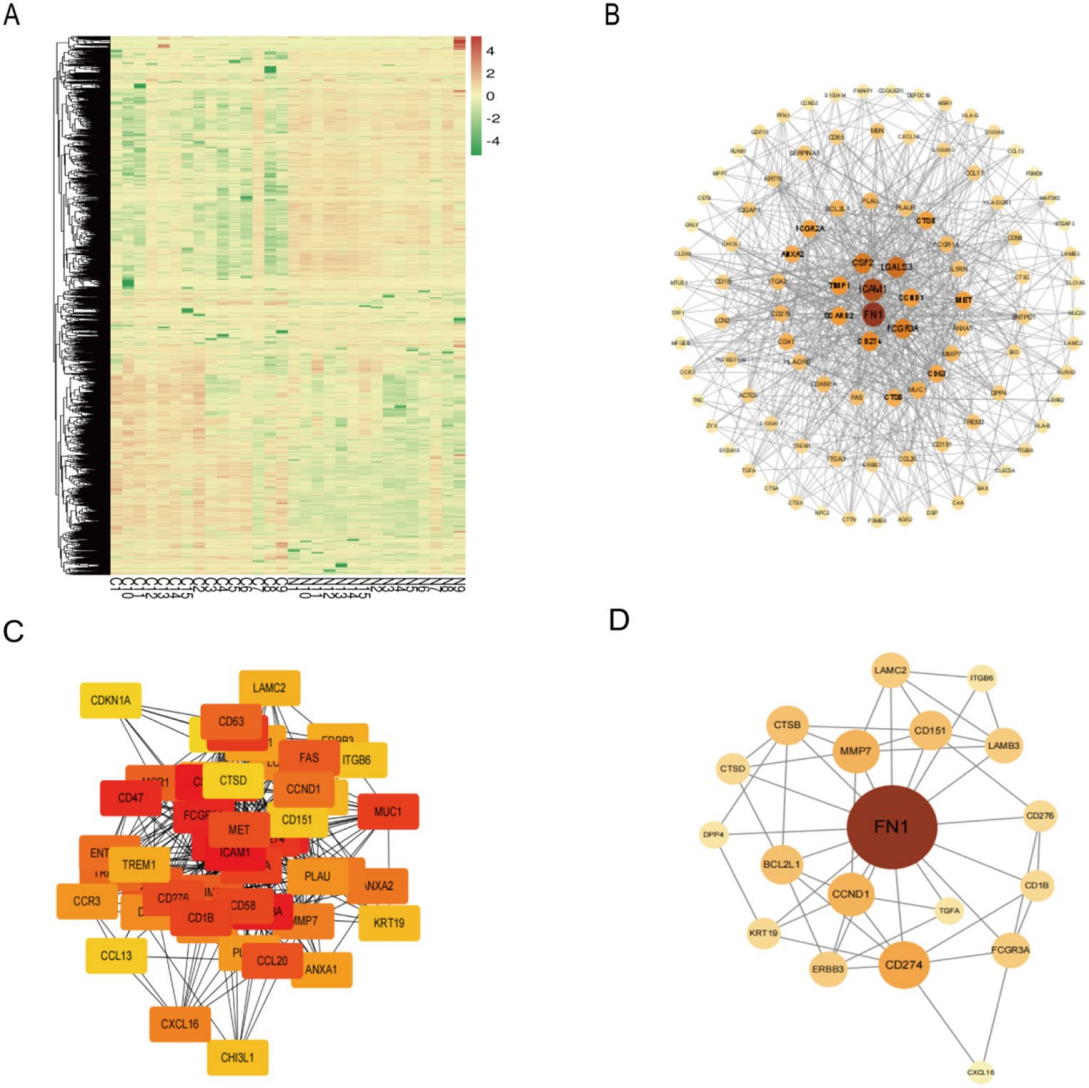
Screening of DEGs in human PTC tissues and adjacent non-cancerous tissues by ONT sequencing. A. Heatmap of all DEGs in cancer versus adjacent non-cancerous tissues from patients with confirmed PTC. B-D. PPI network of the top 100 and top 50 critical DEGs designed by Cytoscape

To explore novel molecular markers of PTC, we collected cancer versus adjacent non-cancerous tissues from 15 patients with confirmed PTC for ONT sequencing and found a total of 2,147 DEGs, and constructed a heat map (Fig. 10A), in which 1047 genes tended to be significantly upregulated. Screening was then performed according to | log (Fold Change (FC)) | ≥ 1.5 and *P* < 0.05, and the top 500 up-regulated DEGs have been selected to build the total PPI network, and the top 100 key Hub genes were found to construct the sub-PPI network by Cytohubba (Fig. 10B). The subnetwork in which KRT19 was located was screened by Mcode (Fig. 10C), and KRT19 was found to be located in the top 50 critical Hub genes. The associated PPI network was again drawn for the top 50 critical Hub genes (Fig. 10D). Critical DEGs were ranked as ICAM1, CSF2, FCGR3A, FN1, LGALS3, FCGR2A, CD47, SCARB2, CD274, ITGA2, MUC1, FCGR1A, CD1B, CD58, CD276, MET, CCL20, CCL17, FAS, TIMP1, MSR1, CD63, TREM2, ENTPD1, CCND1, ANXA2, IL1RN, MMP7, CXCL16, CTSB, LCN2, DPP4, PLAU, CCR3, PLAUR, ANXA1, BCL2L1, ITGA3, TREM1, ERBB3, LAMC2, LAMB3, ***KRT19***, CHI3L1, ITGB6, CD151, CCL13, CTSD, CDKN1A, TGFA. ***The Log2FC = 3.36 andP = 5.22e-27 of KRT19.***

### Gene ontology (GO) analysis of significantly DEGs

**Fig. 11:**
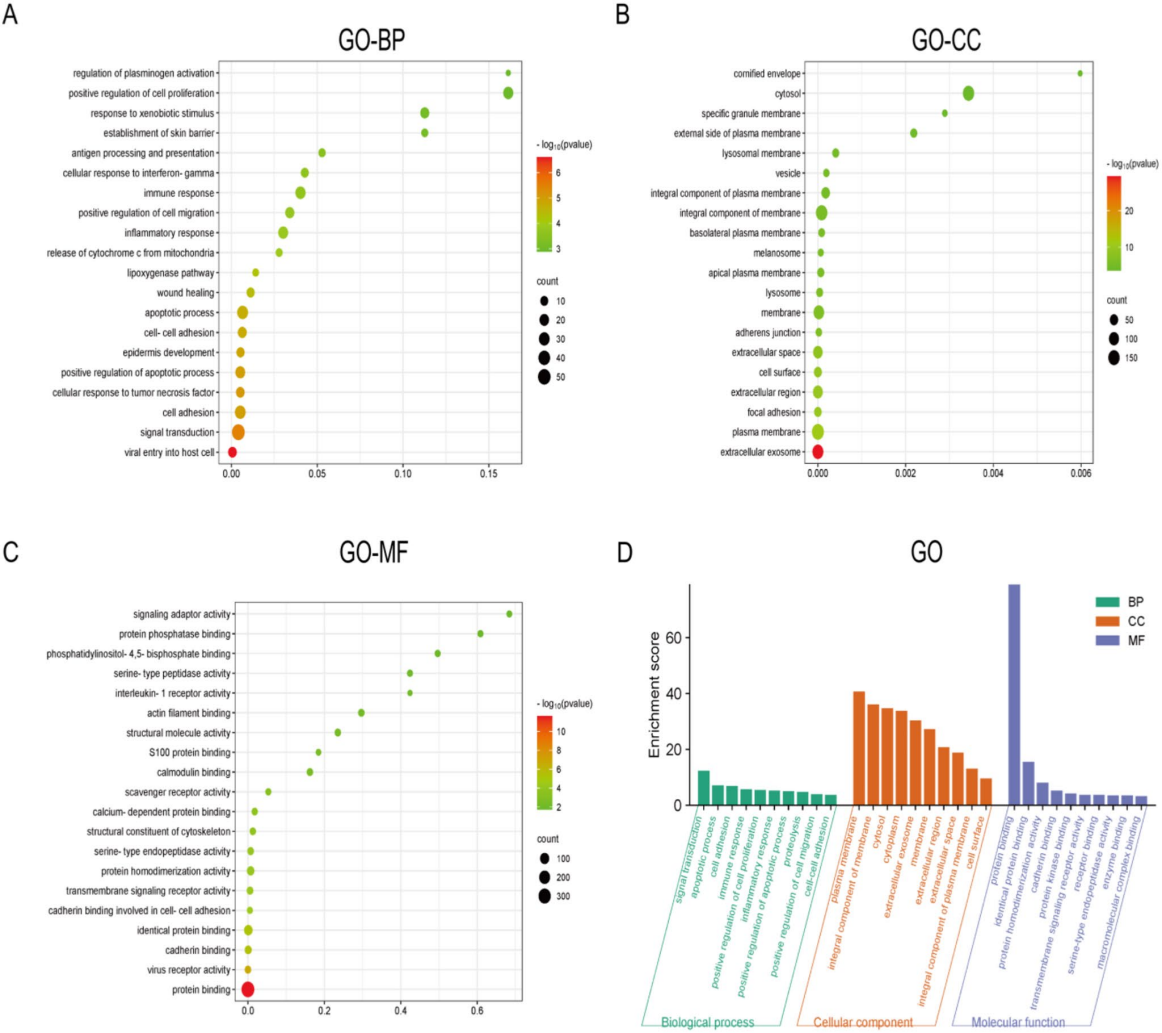
Gene ontology (GO) Analysis of significantly DEGs. The GO analysis results showed that (**A**) the main enriched biological processes included Regulation of plasminogen activation, Positive regulation of cell proliferation, Response to xenobiotic stimulation, etc.; (**B**) the main enriched cellular composition included Cornified envelope, Cytosol, Specific granule membrane, etc.; (**C**) the main enriched molecular functions included adaptor activity, Protein Signaling, Phosphatidylinositol-4, 5-biophosphate binding, etc. (**D**) Histogram of 3-in-1 GO analysis of the first 500 significantly upregulated DEGs

In order to reveal the molecular mechanism of action that promotes the development of PTC, we performed GO functional enrichment analysis on the top 500 significantly up-regulated DEGs screened with the above methods, respectively. The results of GO functional enrichment analysis (Fig. 11D) showed that among the top 500 significantly up-regulated DEGs, the main enrichment was in biological process terms including Regulation of plasminogen activation, Positive regulation of cell proliferation, and Response to xenobiotic stimulus (Fig. 11A). In addition, significant enrichment in terms of cellular component was mainly associated with: Cornified envelope, Cytosol, Specific granule membrane (Fig. 11B). Finally, significant enrichment in molecular function terms included: Signaling adaptor activity, Protein phosphatase binding, Phosphatidylinositol-4,5-biophosphate binding (Fig. 11C).

### ESR1, KRT19 interactions with upstream and downstream proteins

**Fig. 12:**
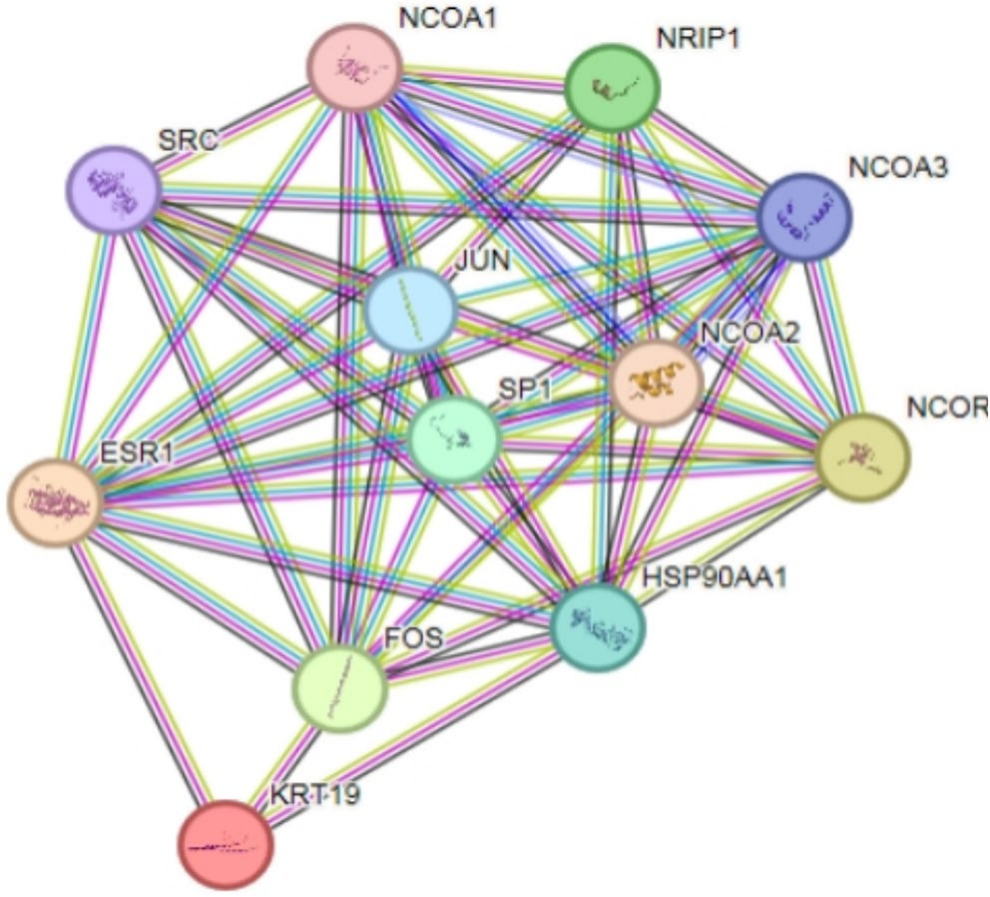
ESR1, KRT19 protein interaction network diagram

In the STRING website, analyzing the relationship between ESR1, KRT19 and upstream and downstream proteins, the corresponding relationship network diagram can be obtained(Fig. 12). PPI enrichment *p*-value = 2.93e-07, containing a total of 12 nodes such as FOS and HSP90AA1 interacting with ESR1 and KRT19, of which ESR1 interacts with KRT19 (score = 0.59). ESR1 and KRT19 are mainly involved in the Cellular response to prolactin、Cellular response to Thyroglobulin triiodothyronine、Positive regulation of adipose tissue development and other biological processes (Additional file: Table S1); mainly involved in Nitric-oxide synthase regulator activity、Aryl hydrocarbon receptor binding、Nuclear estrogen receptor binding and other molecular functions (Table 1); and mainly involved in Transcription factor AP-1 complex、Dendritic growth cone、Euchromatin and other cellular components (Additional file: Table S2). ESR1 and KRT19 are mainly involved in KEGG pathways such as the Estrogen signaling pathway、Endocrine resistance、Thyroid hormone signaling pathway (Table 2).

**Table 1:** Top5 of ESR1、KRT19 molecular functions

GO ID	Functional Description	Number of enriched differential genes/ Total number of genes	*P* values
GO:0030235	Nitric-oxide synthase regulator activity	2 /8	0.0054
GO:0017162	Aryl hydrocarbon receptor binding	2 /9	0.0054
***GO:0030331***	***Nuclear estrogen receptor binding***	***4 /41***	***1.14e-05***
GO:0070412	R-SMAD binding	2 /24	0.02
GO:0046966	Nuclear thyroid hormone receptor binding	2 /29	0.0249

**Table 2:** Top5 of ESR1、KRT19 KEGG signaling pathways

Pathway	Name	Number of enriched differential genes/ Total number of genes	*P* values
***hsa04915***	***Estrogen signaling pathway***	***10 /133***	***6.43e-18***
hsa01522	Endocrine resistance	7 /94	9.78e-12
hsa04919	Thyroid hormone signaling pathway	6 /120	6.07e-09
hsa04137	Mitophagy - animal	3 /64	0.00039
hsa04917	Prolactin signaling pathway	3 /68	0.0004

## Discussion

In this study, we explored the mechanism of estrogen on the occurrence and development of PTC from the epidemiological findings that the high estrogen level in women of childbearing age is a clinical phenomenon that promotes them to become a population with a high incidence of PTC. Estrogen action is mainly mediated by its receptors ERα (ESR1) and ERβ (ESR2) [30]. In the literature, ERs are nuclear receptors, and ERα exerts cancer-promoting effects through different molecular mechanisms in a variety of malignancies, including PTC, while ERβ mainly exerts protective effect [31–33]. So we chose to focus our eyes on ERα. Through Ualcan online analysis of ESR1 and KRT19 in the TCGA database, it was found that both of them had elevated expression in THCA compared with normal thyroid tissue, and it was considered that both ESR1 and KRT19 may be involved in the pathological process of THCA. This suggests that we use KRT19 as a potential target gene to investigate the estrogen pathogenesis of PTC and conduct an in-depth study of the relationship between ESR1 and KRT19 and the development of PTC.

Our in vivo results showed that high E2 levels in vivo promoted the growth and proliferation of PTC xenograft tissues in mice, which again verified that estrogen is one of the triggers of PTC. Next, through in vivo and in vitro experiments, it was verified that E2 promoted the proliferation and growth of PTC cells and PTC xenograft tissues through the activation of ERα.

Combined with the previous results that KRT19 was significantly differentially expressed in THCA by Ualcan online analysis of the TCGA database, in vitro experiments verified that KRT19 was overexpressed in PTC cells compared with normal human thyroid epithelial cells. Using cell transfection technique, immunofluorescence and CCK8 showed that the proliferation level of PTC cells decreased after knockdown of KRT19; Western blot assay was used to detect the expression level of EMT-related proteins again, combined with cell scratch and transwell assay demonstrated that the migration and invasion ability of PTC cells were also significantly reduced after knockdown of KRT19. Thus, we deeply explored the role of KRT19 on PTC function based on public databases and demonstrated that high expression of KRT19 in PTC promotes tumor growth and metastasis. We believe that KRT19 can be used as a PTC molecular marker for the diagnosis and metastasis.

E2 activation of ERα as well as overexpression of KRT19 both showed a positive trend towards growth and metastasis of PTC which aroused our attention. However, in PTC, the molecular role relationship between ERα and KRT19 is unclear. Therefore, we focused more on exploring the relationship between ERα and KRT19 in the following experiments. The protein expression levels and immunofluorescence staining of ERα and KRT19 in different groups were examined by Western blot. The results showed that ERα and KRT19 had a relationship, and they constituted the ERα/KRT19 unidirectional signaling axis. Finally, to investigate the effect of ERα/KRT19 signaling axis in estrogen-promoted PTC development, we again revealed through in vitro experiments that E2 promoted the proliferation, migration and invasion of PTC cells through activation of ERα/KRT19 signaling axis. This finding preliminarily reveals the specific mechanism of KRT19 as a marker in promoting the development of PTC; explores a novel molecular mechanism of estrogen in PTC.

To demonstrate the public database and our experimental results, we screened and analyzed the bioinformatics results of ONT sequencing from 15 pairs of human PTC tissues and adjacent non-cancerous tissues previously collected by our group. By constructing the PPI network, KRT19 was found in the top 50 significantly upregulated DEGs. It again reveals the feasibility of KRT19 as a PTC molecular marker for the clinical diagnosis. The STRING website reports that ESR1 does have a protein interaction with KRT19. The results enriched in both KEGG and GO analyses were associated with estrogen signaling pathway and estrogen receptor binding. The accuracy of our previous findings on the molecular mechanism of action of estrogen in PTC was verified by these bioinformatics analysis methods.

## Conclusions

We discovered molecular indicators associated with PTC screening and diagnosis, and we corroborated the clinical perspective that estrogen is one of the causal factors of PTC by starting with public databases and clinical ONT sequencing. Also, we deeply explored the molecular mechanism of estrogen action in PTC and concluded that estrogen promotes the proliferation, migration and invasion of PTC by activating the ERα/KRT19 signaling axis. Our study provides a new idea for clinical diagnosis and treatment.

## Electronic supplementary material

Below is the link to the electronic supplementary material.

Supplementary Material 1

Supplementary Material 2

## Data Availability

The datasets used and/or analyzed during the current study are available from the corresponding author on reasonable request. We have uploaded RNA-seq data mentioned in the manuscript to the NCBI database but this data is not currently publicly available; BioProject accession: PRJNA1033723 RNA sequencing (https://dataview.ncbi.nlm.nih.gov/object/PRJNA1033723?reviewer=b7u9pa0cddc5cb6sl8p7hsn86g).

## Abbreviations

THCA: Thyroid cancer
PTC: Papillary thyroid carcinoma
ERs: Estrogen receptors
ERα: Estrogen receptor α
KRT19: Keratin 19
E2: β-estradiol
ONT: Oxford Nanopore Technologies
DEG: Differentially expressed gene
FC: Fold change
PPI: Protein-protein interaction
GO: Gene ontology
KEGG: Kyoto Encyclopedia of Genes and Genomes

## References

[1] Rahib L, Smith BD, Aizenberg R, Rosenzweig AB, Fleshman JM, Matrisian LM (2014) Projecting cancer incidence and deaths to 2030: the unexpected burden of thyroid, liver, and pancreas cancers in the United States. Cancer Res 74(11):2913–2921. 10.1158/0008-5472.Can-14-0155 24840647 10.1158/0008-5472.CAN-14-0155

[2] Schneider DF, Chen H (2013) New developments in the diagnosis and treatment of thyroid cancer. CA Cancer J Clin 63(6):374–394. 10.3322/caac.21195 23797834 10.3322/caac.21195 PMC3800231

[3] Yip J, Orlov S, Orlov D, Vaisman A, Hernández KG, Etarsky D, Kak I, Parvinnejad N, Freeman JL, Walfish PG (2013) Predictive value of metastatic cervical lymph node ratio in papillary thyroid carcinoma recurrence. Head Neck 35(4):592–598. 10.1002/hed.23047 22730192 10.1002/hed.23047

[4] Zane M, Parello C, Pennelli G, Townsend DM, Merigliano S, Boscaro M, Toniato A, Baggio G, Pelizzo MR, Rubello D et al (2017) Estrogen and thyroid cancer is a stem affair: a preliminary study. Biomed Pharmacother 85:399–411. 10.1016/j.biopha.2016.11.043 27899250 10.1016/j.biopha.2016.11.043 PMC5218826

[5] Rahbari R, Zhang L, Kebebew E (2010) Thyroid cancer gender disparity. Future Oncol 6(11):1771–1779. 10.2217/fon.10.127 21142662 10.2217/fon.10.127 PMC3077966

[6] Dong W, Zhang H, Li J, Guan H, He L, Wang Z, Shan Z, Teng W (2013) Estrogen induces metastatic potential of papillary thyroid Cancer cells through estrogen receptor α and β. Int J Endocrinol 2013:941568. 10.1155/2013/941568 24222765 10.1155/2013/941568 PMC3810507

[7] Rajoria S, Suriano R, Shanmugam A, Wilson YL, Schantz SP, Geliebter J, Tiwari RK (2010) Metastatic phenotype is regulated by estrogen in thyroid cells. Thyroid 20(1):33–41. 10.1089/thy.2009.0296 20067378 10.1089/thy.2009.0296 PMC2833180

[8] Mangelsdorf DJ, Thummel C, Beato M, Herrlich P, Schütz G, Umesono K, Blumberg B, Kastner P, Mark M, Chambon P et al (1995) The nuclear receptor superfamily: the second decade. Cell 83(6):835–839. 10.1016/0092-8674(95)90199-x 8521507 10.1016/0092-8674(95)90199-x PMC6159888

[9] Derwahl M, Nicula D (2014) Estrogen and its role in thyroid cancer. Endocr Relat Cancer 21(5):T273–283. 10.1530/erc-14-0053 25052473 10.1530/ERC-14-0053

[10] Manole D, Schildknecht B, Gosnell B, Adams E, Derwahl M (2001) Estrogen promotes growth of human thyroid tumor cells by different molecular mechanisms. J Clin Endocrinol Metab 86(3):1072–1077. 10.1210/jcem.86.3.7283 11238488 10.1210/jcem.86.3.7283

[11] Lee ML, Chen GG, Vlantis AC, Tse GM, Leung BC, van Hasselt CA (2005) Induction of thyroid papillary carcinoma cell proliferation by estrogen is associated with an altered expression of Bcl-xL. Cancer J 11(2):113–121. 10.1097/00130404-200503000-00006 15969986 10.1097/00130404-200503000-00006

[12] Xue L, Yan H, Chen Y, Zhang Q, Xie X, Ding X, Wang X, Qian Z, Xiao F, Song Z et al (2019) EZH2 upregulation by ERα induces proliferation and migration of papillary thyroid carcinoma. BMC Cancer 19(1):1094. 10.1186/s12885-019-6306-9 31718595 10.1186/s12885-019-6306-9 PMC6852908

[13] Heikkilä A, Hagström J, Mäenpää H, Louhimo J, Siironen P, Heiskanen I, Haglund C, Arola J (2013) Loss of estrogen receptor Beta expression in follicular thyroid carcinoma predicts poor outcome. Thyroid 23(4):456–465. 10.1089/thy.2012.0363 23106428 10.1089/thy.2012.0363

[14] Halon A, Nowak-Markwitz E, Maciejczyk A, Pudelko M, Gansukh T, Györffy B, Donizy P, Murawa D, Matkowski R, Spaczynski M et al (2011) Loss of estrogen receptor beta expression correlates with shorter overall survival and lack of clinical response to chemotherapy in ovarian cancer patients. Anticancer Res 31(2):711–718 21378361

[15] Fu X, Sun X, Li X, Sheng Z (2001) Dedifferentiation of epidermal cells to stem cells in vivo. Lancet 358(9287):1067–1068. 10.1016/s0140-6736(01)06202-x 11589942 10.1016/S0140-6736(01)06202-X

[16] Yuan X, Yi M, Dong B, Chu Q, Wu K (2021) Prognostic significance of KRT19 in lung squamous Cancer. J Cancer 12(4):1240–1248. 10.7150/jca.51179 33442422 10.7150/jca.51179 PMC7797641

[17] Asfaha S, Hayakawa Y, Muley A, Stokes S, Graham TA, Ericksen RE, Westphalen CB, von Burstin J, Mastracci TL, Worthley DL et al (2015) Krt19(+)/Lgr5(-) cells are Radioresistant Cancer-initiating stem cells in the Colon and intestine. Cell Stem Cell 16(6):627–638. 10.1016/j.stem.2015.04.013 26046762 10.1016/j.stem.2015.04.013 PMC4457942

[18] Sun Z, Zhou R, Dai J, Chen J, Liu Y, Wang M, Zhou R, Liu F, Zhang Q, Xu Y et al (2023) KRT19 is a Promising Prognostic Biomarker and Associates with Immune infiltrates in Serous Ovarian Cystadenocarcinoma. Int J Gen Med 16:4849–4862. 10.2147/ijgm.S419235 37916194 10.2147/IJGM.S419235 PMC10616674

[19] Saha SK, Choi HY, Kim BW, Dayem AA, Yang GM, Kim KS, Yin YF, Cho SG (2017) KRT19 directly interacts with β-catenin/RAC1 complex to regulate NUMB-dependent NOTCH signaling pathway and breast cancer properties. Oncogene 36(3):332–349. 10.1038/onc.2016.221 27345400 10.1038/onc.2016.221 PMC5270332

[20] Wang X, Xu X, Peng C, Qin Y, Gao T, Jing J, Zhao H (2019) BRAF(V600E)-induced KRT19 expression in thyroid cancer promotes lymph node metastasis via EMT. Oncol Lett 18(1):927–935. 10.3892/ol.2019.10360 31289571 10.3892/ol.2019.10360 PMC6539636

[21] Wa Kammal WS, Yahaya A, Shah SA, Abdullah Suhaimi SN, Mahasin M, Mustangin M, Md Isa N (2019) The diagnostic utility of cytokeratin 19 in differentiating malignant from benign thyroid lesions. Malays J Pathol 41(3):293–301 31901914

[22] Hong S, Xie Y, Cheng Z, Li J, He W, Guo Z, Zhang Q, Peng S, He M, Yu S et al (2022) Distinct molecular subtypes of papillary thyroid carcinoma and gene signature with diagnostic capability. Oncogene 41(47):5121–5132. 10.1038/s41388-022-02499-0 36253446 10.1038/s41388-022-02499-0 PMC9674518

[23] Prasad PA, Raju K (2022) Diagnostic utility of CK19 and galectin-3 in differentiating papillary thyroid carcinoma from nonneoplastic lesions of thyroid. J Cancer Res Ther 18(3):644–649. 10.4103/jcrt.jcrt_563_21 35900535 10.4103/jcrt.jcrt_563_21

[24] Huang Y, Prasad M, Lemon WJ, Hampel H, Wright FA, Kornacker K, LiVolsi V, Frankel W, Kloos RT, Eng C et al (2001) Gene expression in papillary thyroid carcinoma reveals highly consistent profiles. Proc Natl Acad Sci U S A 98(26):15044–15049. 10.1073/pnas.251547398 11752453 10.1073/pnas.251547398 PMC64980

[25] Luqmani YA, Al Azmi A, Al Bader M, Abraham G, El Zawahri M (2009) Modification of gene expression induced by siRNA targeting of estrogen receptor alpha in MCF7 human breast cancer cells. Int J Oncol 34(1):231–242 19082494

[26] Chandrashekar DS, Bashel B, Balasubramanya SAH, Creighton CJ, Ponce-Rodriguez I, Chakravarthi B, Varambally S (2017) UALCAN: a portal for facilitating Tumor Subgroup Gene expression and survival analyses. Neoplasia 19(8):649–658. 10.1016/j.neo.2017.05.002 28732212 10.1016/j.neo.2017.05.002 PMC5516091

[27] Szklarczyk D, Gable AL, Nastou KC, Lyon D, Kirsch R, Pyysalo S, Doncheva NT, Legeay M, Fang T, Bork P et al (2021) The STRING database in 2021: customizable protein-protein networks, and functional characterization of user-uploaded gene/measurement sets. Nucleic Acids Res 49(D1):D605–d612. 10.1093/nar/gkaa1074 33237311 10.1093/nar/gkaa1074 PMC7779004

[28] Jiang C, Xu F, Yi D, Jiang B, Wang R, Wu L, Ding H, Qin J, Lee Y, Sang J et al (2024) Testosterone promotes the migration, invasion and EMT process of papillary thyroid carcinoma by up-regulating Tnnt1. J Endocrinol Invest 47(1):149–166. 10.1007/s40618-023-02132-1 37477865 10.1007/s40618-023-02132-1 PMC10776714

[29] Sherman BT, Hao M, Qiu J, Jiao X, Baseler MW, Lane HC, Imamichi T, Chang W (2022) DAVID: a web server for functional enrichment analysis and functional annotation of gene lists (2021 update). Nucleic Acids Res 50(W1):W216–w221. 10.1093/nar/gkac194 35325185 10.1093/nar/gkac194 PMC9252805

[30] Katzenellenbogen BS (2021) Estrogen receptor gets a grip on RNA. Cell 184(20):5086–5088. 10.1016/j.cell.2021.09.012 34559987 10.1016/j.cell.2021.09.012

[31] Zhou H, Xie X, Chen Y, Lin Y, Cai Z, Ding L, Wu Y, Peng Y, Tang S, Xu H (2020) Chaperone-mediated Autophagy governs progression of papillary thyroid carcinoma via PPARγ-SDF1/CXCR4 signaling. J Clin Endocrinol Metab 105(10). 10.1210/clinem/dgaa366 10.1210/clinem/dgaa366 32556197

[32] Lung DK, Reese RM, Alarid ET (2020) Intrinsic and extrinsic factors governing the transcriptional regulation of ESR1. Horm Cancer 11(3–4):129–147. 10.1007/s12672-020-00388-0 32592004 10.1007/s12672-020-00388-0 PMC7384552

[33] Yang S, Gong Z, Liu Z, Wei M, Xue L, Vlantis AC, Zhang Y, Chan JY, van Hasselt CA, Zeng X et al (2021) Differential effects of Estrogen Receptor Alpha and Beta on endogenous ligands of peroxisome proliferator-activated receptor gamma in papillary thyroid Cancer. Front Endocrinol (Lausanne) 12708248. 10.3389/fendo.2021.708248 10.3389/fendo.2021.708248 PMC8453163 34557159

