# Supplementary material for: Estrogen enhances the proliferation, migration, and invasion of papillary thyroid carcinoma via the ERα/KRT19 signaling axis
Source: J Endocrinol Invest. 2024 Oct 25;48(3):653–70. doi: 10.1007/s40618-024-02473-5 (PMC11876195; doi:10.1007/s40618-024-02473-5)
Supplement: Supplementary file 1 — Supplementary Material 1 [file 40618_2024_2473_MOESM1_ESM.docx]

Additional file

Table S1 Top5 of ESR1、KRT19 biological processes

| GO ID | Functional Description | Number of enriched differential genes/ Total number of genes | P values |
| --- | --- | --- | --- |
| GO:1990646 | Cellular response to prolactin | 2 /2 | 0.00089 |
| GO:1904017 | Cellular response to Thyroglobulin triiodothyronine | 2 /3 | 0.0013 |
| GO:1904179 | Positive regulation of adipose tissue development | 2 /8 | 0.0042 |
| GO:0035357 | Peroxisome proliferator activated receptor signaling pathway | 2 /9 | 0.005 |
| GO:0045475 | Locomotor rhythm | 2 /12 | 0.0072 |

Table S2 Top5 of ESR1、KRT19 cellular components

| GO ID | Functional Description | Number of enriched differential genes/ Total number of genes | P values |
| --- | --- | --- | --- |
| GO:0035976 | Transcription factor AP-1 complex | 2 /5 | 0.0017 |
| GO:0044294 | Dendritic growth cone | 2 /9 | 0.0025 |
| GO:0000791 | Euchromatin | 3 /60 | 0.0017 |
| GO:0017053 | Transcription repressor complex | 3 /77 | 0.0021 |
| GO:0090575 | RNA polymerase II transcription regulator complex | 4 /256 | 0.0021 |
