# Supplementary material for: Estrogen enhances the proliferation, migration, and invasion of papillary thyroid carcinoma via the ERα/KRT19 signaling axis
Source: J Endocrinol Invest. 2024 Oct 25;48(3):653–70. doi: 10.1007/s40618-024-02473-5 (PMC11876195; doi:10.1007/s40618-024-02473-5)
Supplement: Supplementary file 2 — Supplementary Material 2 [file 40618_2024_2473_MOESM2_ESM.docx]

Fig 4D

**CON E2**


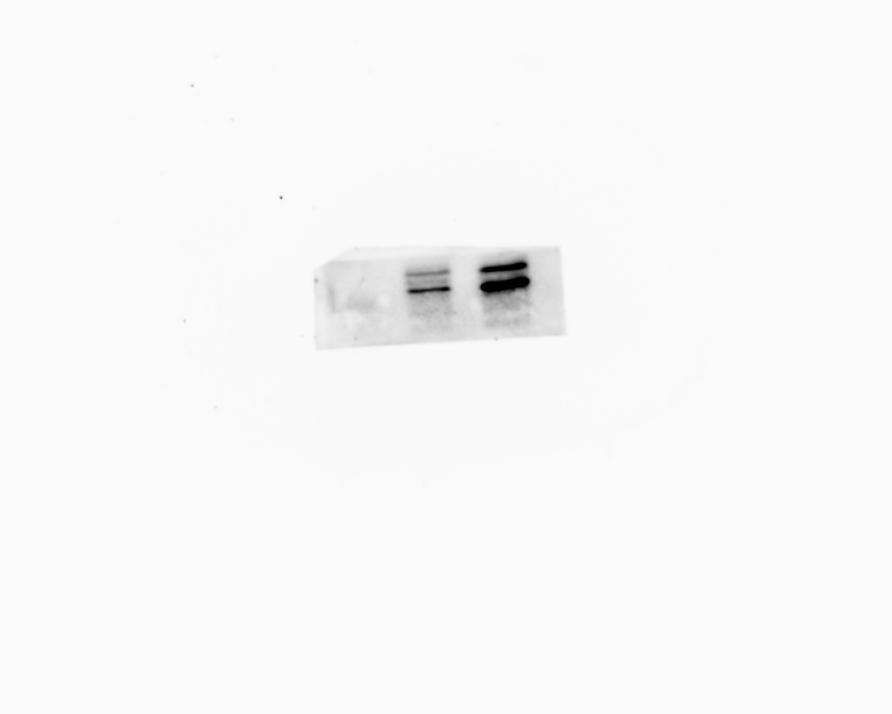


40 KDa

33 KDa

PCNA（36kda）


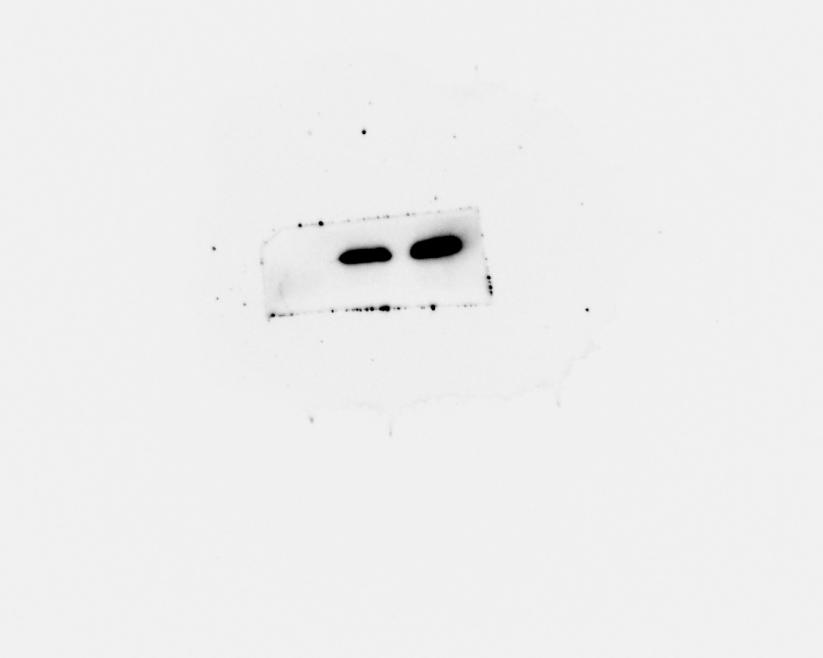


GAPDH（37kda）

（）

33 KDa

Fig 5A


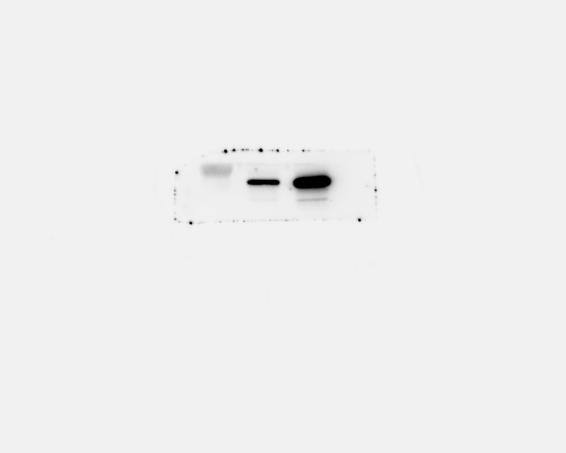


**CON E2**

ERα（66kda）

（）

72 KDa


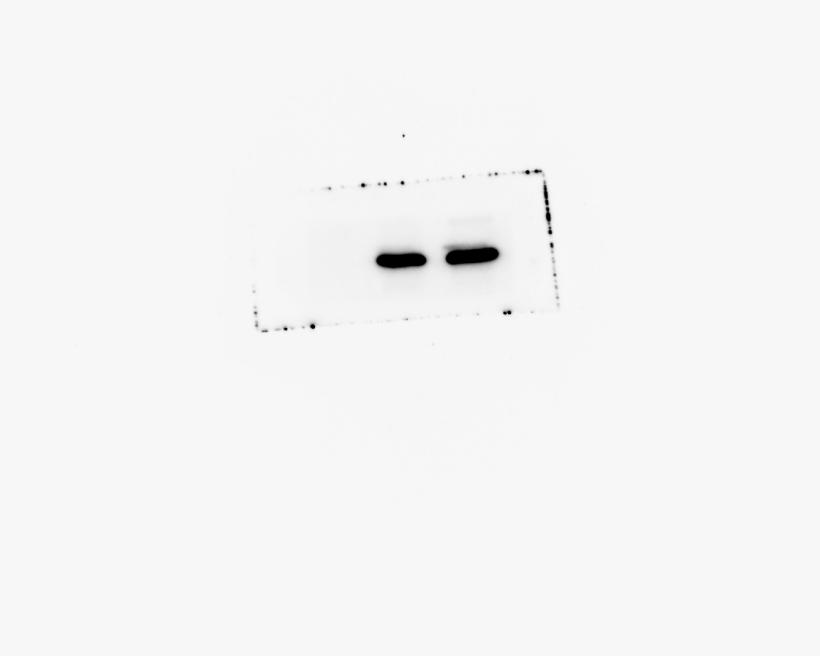


33 KDa

GAPDH（37kda）

（）

Fig 6A


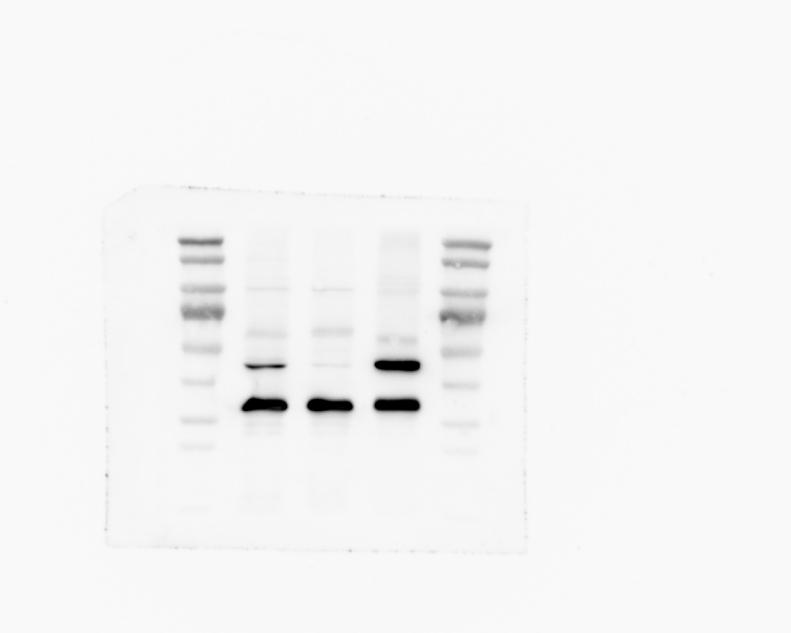


**Nthy TPC-1 KTC-1**

40 KDa

72 KDa

55 KDa

33 KDa

GAPDH（37kda）

（）

KRT19（44kda）

（）

Fig 6B

**CON Si-NC Si-KRT19**


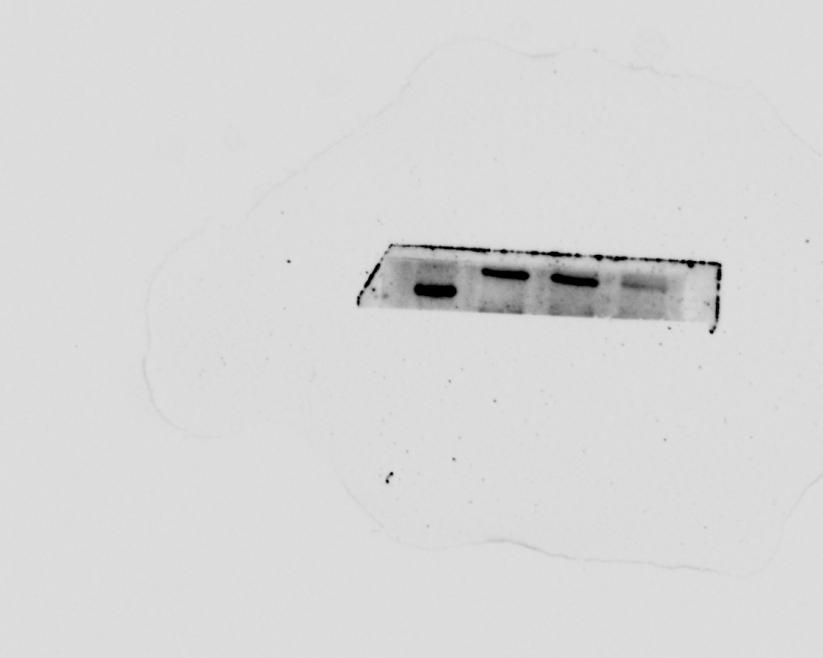


KRT19（44kda）

（）

40 KDa


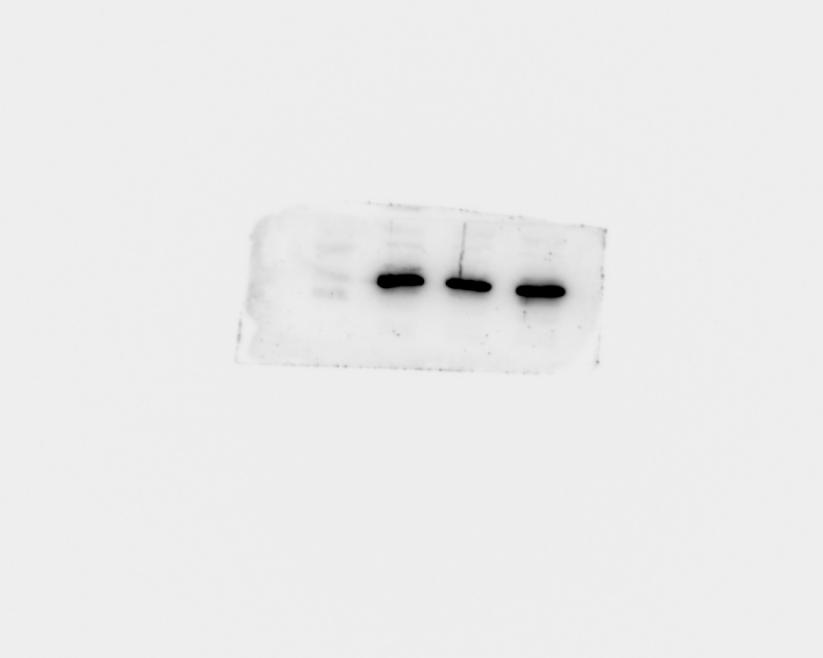


33 KDa

GAPDH（37kda）

（）

Fig 7A

**Si-NC Si-KRT19**


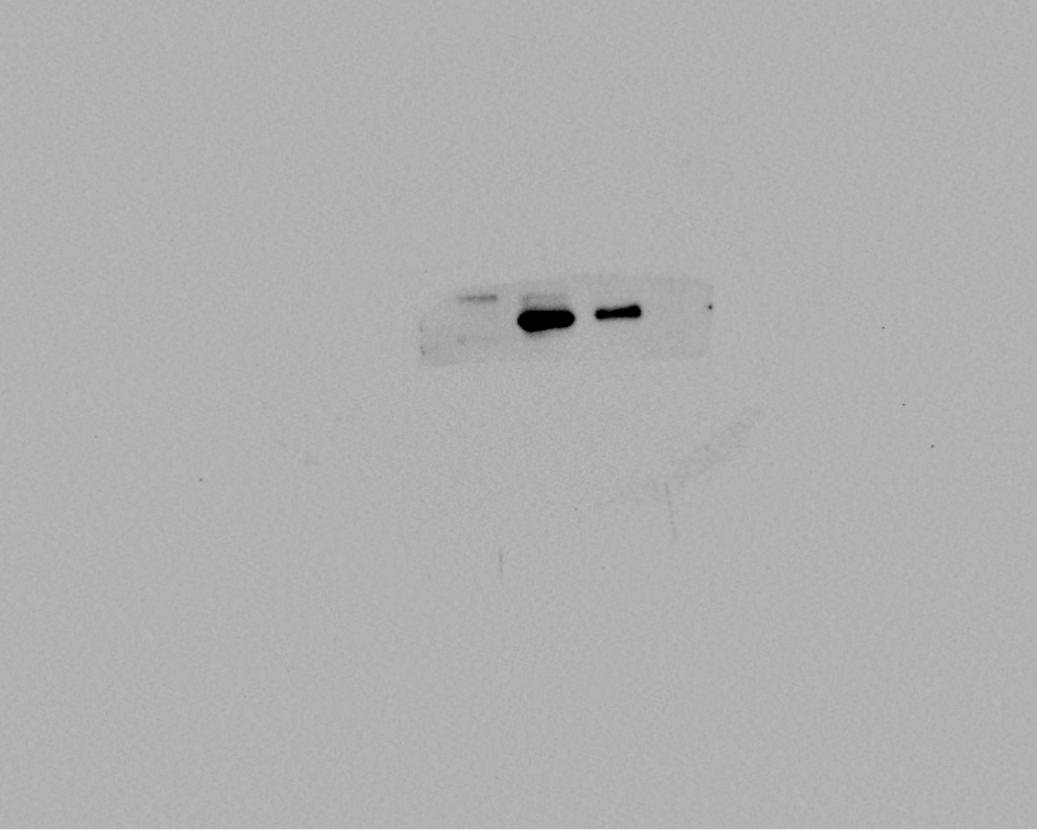


Vimentin（53kda）

（）

55 KDa


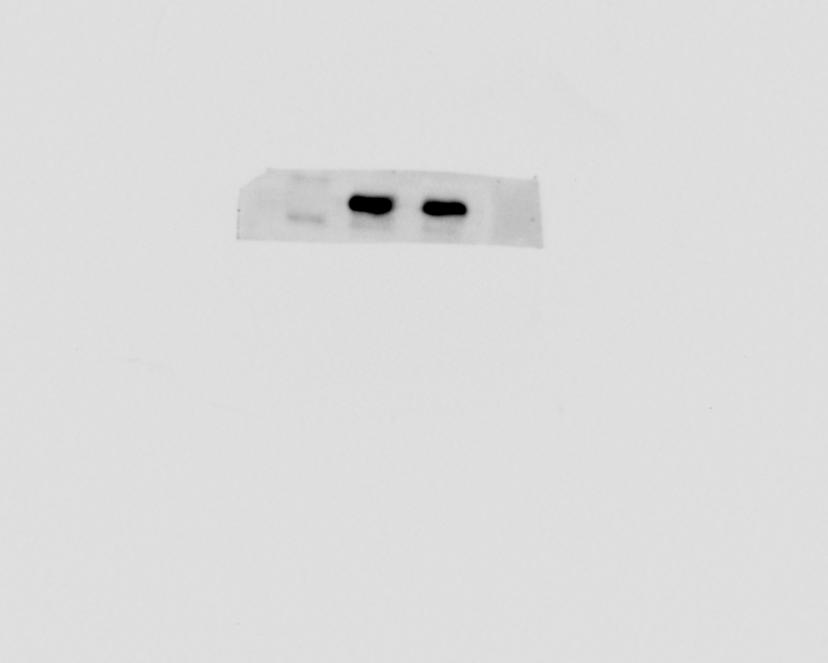


GAPDH（37kda）

（）

33 KDa


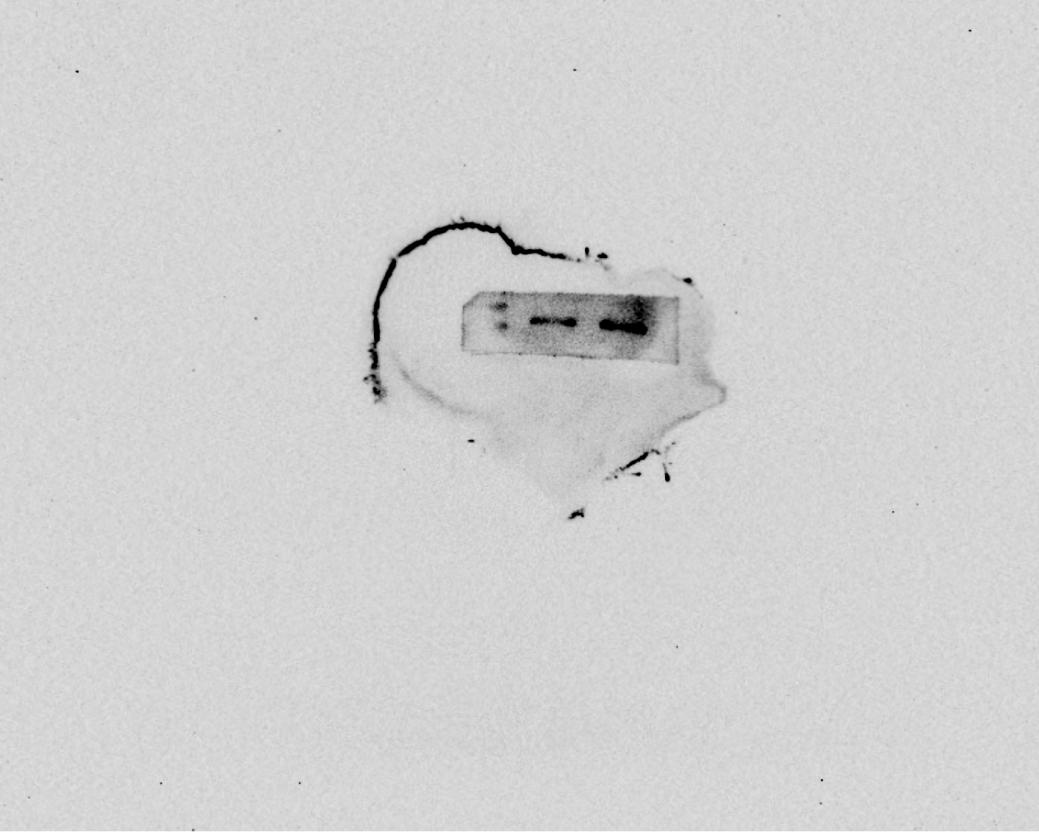


**Si-NC Si-KRT19**

130 KDa

180 KDa

E-cad（135kda）


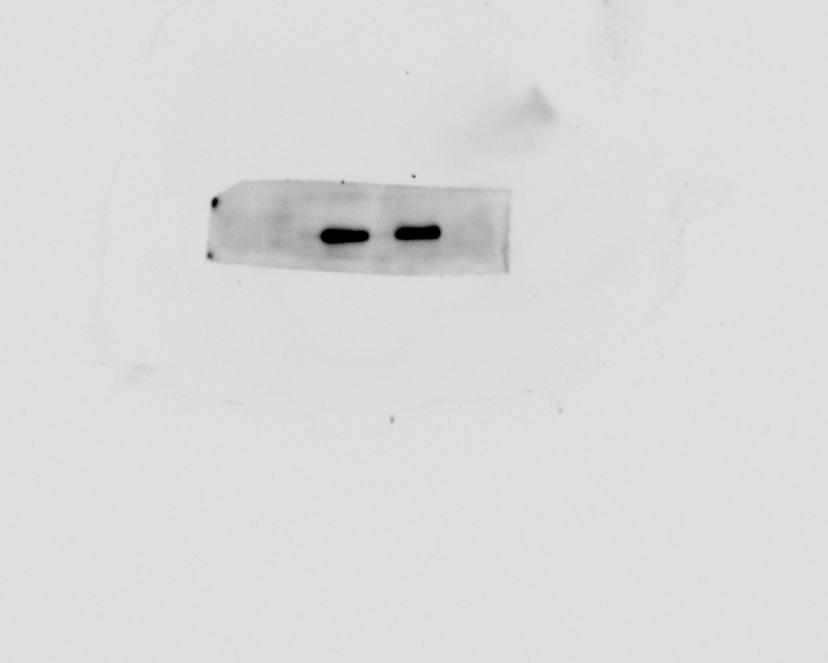


GAPDH（37kda）

（）

40 KDa

33 KDa

（This is merge-GAPDH）
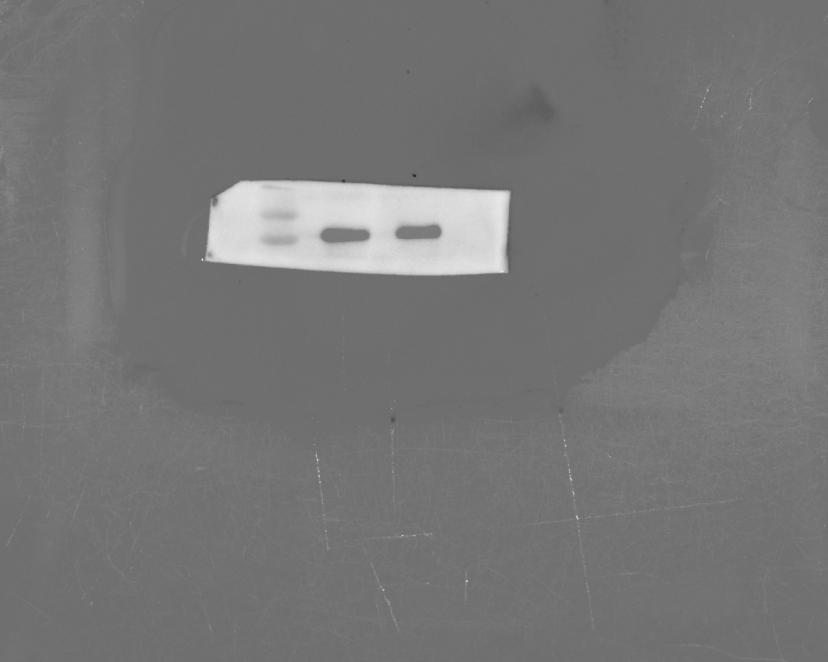


Fig 8A

**Con E2 Mpp**


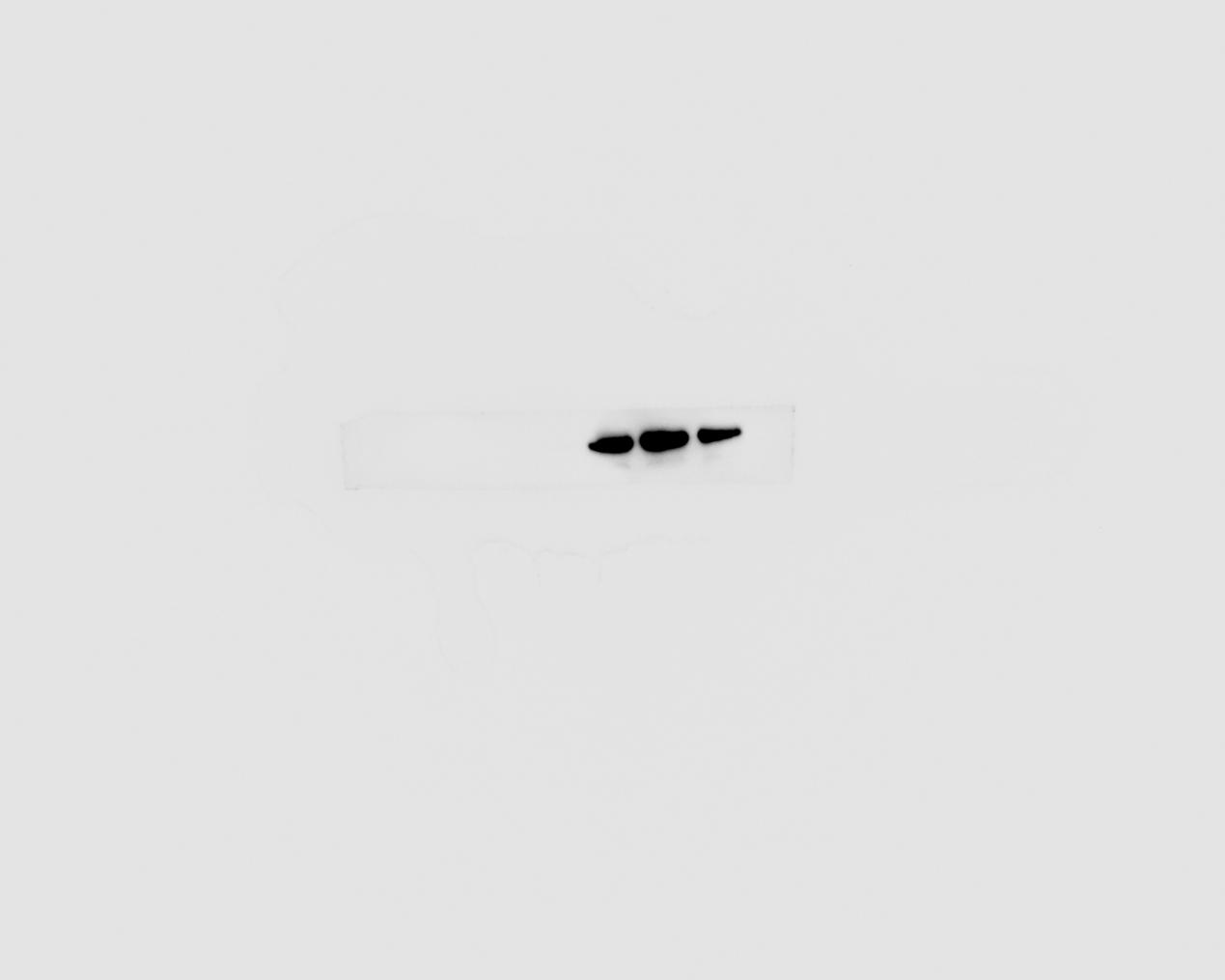


KRT19（44kda）

（）


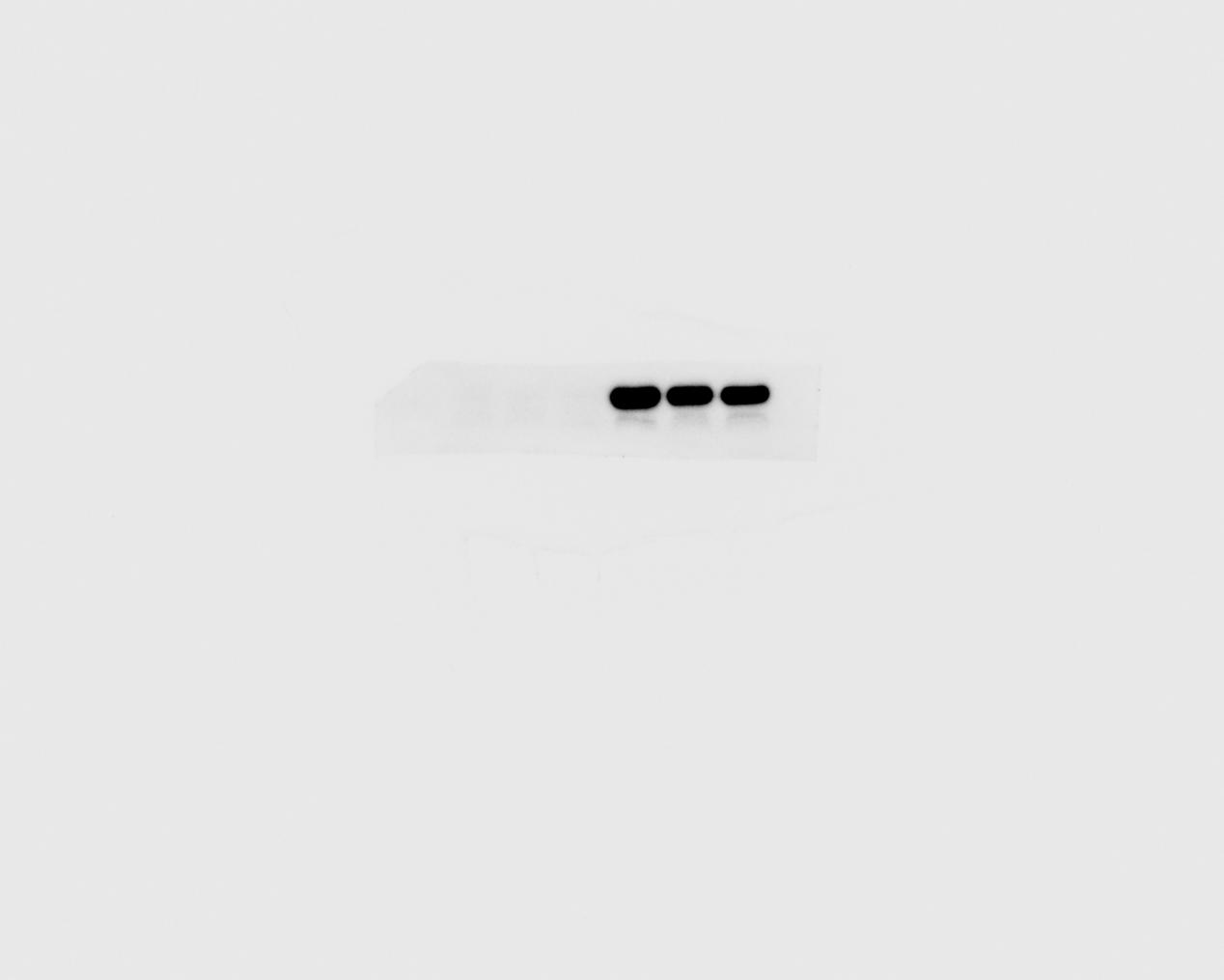


33 KDa

GAPDH（37kda）

（）

Fig8B


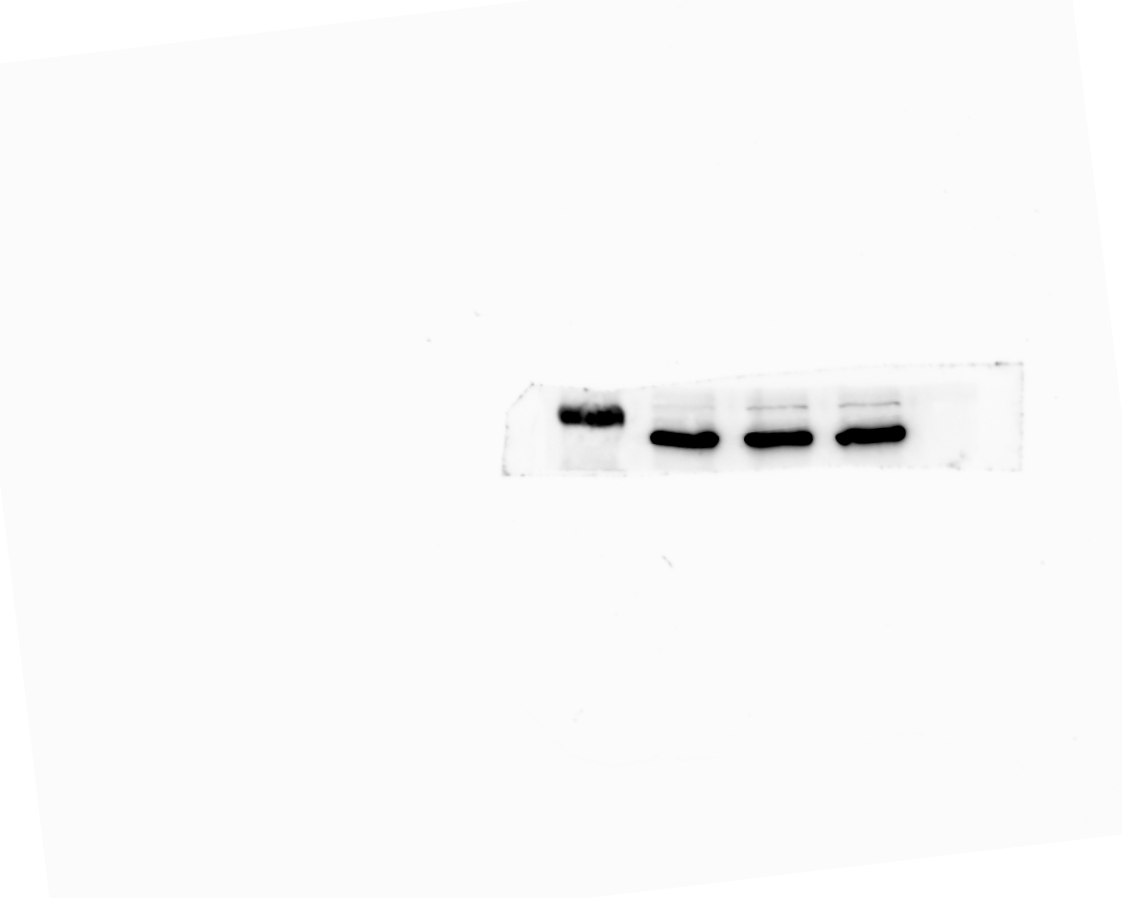


72 KDa

**CON Si-NC Si-KRT19**

ERα（66kda）

（）


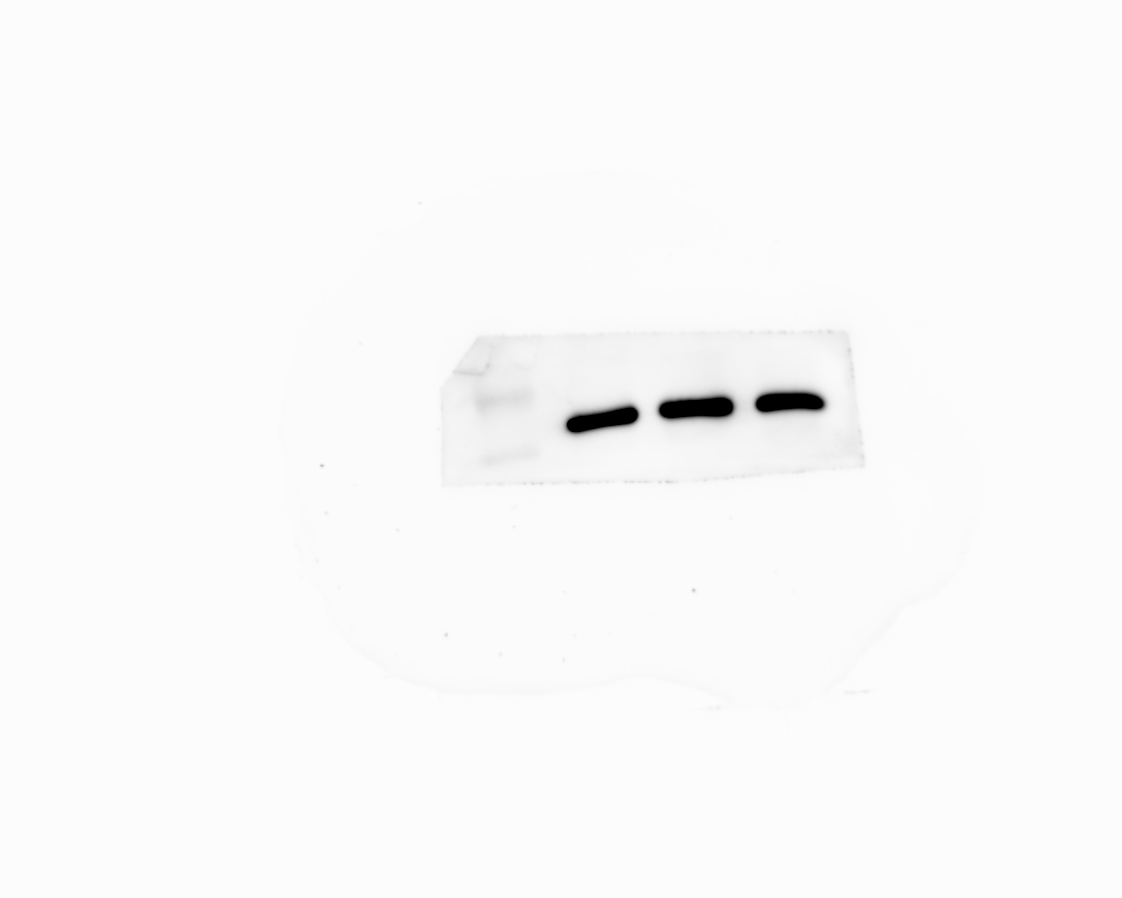


GAPDH（37kda）

（）

33 KDa

40 KDa
